# Integrating single-nucleus RNA sequencing and spatial transcriptomics to elucidate a specialized subpopulation of astrocytes, microglia and vascular cells in brains of mouse model of lipopolysaccharide-induced sepsis-associated encephalopathy

**DOI:** 10.1186/s12974-024-03161-0

**Published:** 2024-07-03

**Authors:** Yanyan Zhu, Yin Zhang, Sheng He, Sanjun Yi, Hao Feng, Xianzhu Xia, Xiaodong Fang, Xiaoqian Gong, Pingsen Zhao

**Affiliations:** 1 Department of Laboratory Medicine, Yuebei People’s Hospital, Shantou University Medical College, No 133, Huimin Road South, Wujiang District, Shaoguan, 512025 China; 2 Laboratory for Diagnosis of Clinical Microbiology and Infection, Yuebei People’s Hospital, Shantou University Medical College, Shaoguan, 512025 China; 3 Research Center for Interdisciplinary & High-quality Innovative Development in Laboratory Medicine, Shaoguan, 512025 China; 4 Shaoguan Municipal Quality Control Center for Laboratory Medicine, Yuebei People’s Hospital, Shantou University Medical College, Shaoguan, 512025 China; 5 Shaoguan Municipal Quality Control Center for Surveillance of Bacterial Resistance, Shaoguan, 512025 China; 6 Shaoguan Engineering Research Center for Research and Development of Molecular and Cellular Technology in Rapid Diagnosis of Infectious Diseases and Cancer, Shaoguan, 512025 China; 7 Yuebei People’s Hospital, Shantou University Medical College, Shaoguan, 512025 China; 8 BGI-Shenzhen, Shenzhen, 518083 China; 9 Jiaxing University Master Degree Cultivation Base, Zhejiang Chinese Medical University, Jiaxing, 314001 China

**Keywords:** Sepsis, SAE, Single-nucleus RNA sequencing, Spatial transcriptomics, *Anxa1*, Glial cell response, Cellular colocalization

## Abstract

**Background:**

Understanding the mechanism behind sepsis-associated encephalopathy (SAE) remains a formidable task. This study endeavors to shed light on the complex cellular and molecular alterations that occur in the brains of a mouse model with SAE, ultimately unraveling the underlying mechanisms of this condition.

**Methods:**

We established a murine model using intraperitoneal injection of lipopolysaccharide (LPS) in wild type and *Anxa1*^*−/−*^ mice and collected brain tissues for analysis at 0-hour, 12-hour, 24-hour, and 72-hour post-injection. Utilizing advanced techniques such as single-nucleus RNA sequencing (snRNA-seq) and Stereo-seq, we conducted a comprehensive characterization of the cellular responses and molecular patterns within the brain.

**Results:**

Our study uncovered notable temporal differences in the response to LPS challenge between Anxa1^−/−^ (annexin A1 knockout) and wild type mice, specifically at the 12-hour and 24-hour time points following injection. We observed a significant increase in the proportion of Astro-2 and Micro-2 cells in these mice. These cells exhibited a colocalization pattern with the vascular subtype Vas-1, forming a distinct region known as V1A2M2, where Astro-2 and Micro-2 cells surrounded Vas-1. Moreover, through further analysis, we discovered significant upregulation of ligands and receptors such as *Timp1-Cd63*, *Timp1-Itgb1*, *Timp1-Lrp1*, as well as *Ccl2-Ackr1* and *Cxcl2-Ackr1* within this region. In addition, we observed a notable increase in the expression of *Cd14-Itgb1*, *Cd14-Tlr2*, and *Cd14-C3ar1* in regions enriched with Micro-2 cells. Additionally, *Cxcl10-Sdc4* showed broad upregulation in brain regions containing both Micro-2 and Astro-2 cells. Notably, upon LPS challenge, there was an observed increase in *Anxa1* expression in the mouse brain. Furthermore, our study revealed a noteworthy increase in mortality rates following *Anxa1* knockdown. However, we did not observe substantial differences in the types, numbers, or distribution of other brain cells between Anxa1^−/−^ and wildtype mice over time. Nevertheless, when comparing the 24-hour post LPS injection time point, we observed a significant decrease in the proportion and distribution of Micro-2 and Astro-2 cells in the vicinity of blood vessels in Anxa1^−/−^ mice. Additionally, we noted reduced expression levels of several ligand-receptor pairs including *Cd14-Tlr2*, *Cd14-C3ar1*, *Cd14-Itgb1*, *Cxcl10-Sdc4*, *Ccl2-Ackr1*, and *Cxcl2-Ackr1*.

**Conclusions:**

By combining snRNA-seq and Stereo-seq techniques, our study successfully identified a distinctive cellular colocalization, referred to as a special pathological niche, comprising Astro-2, Micro-2, and Vas-1 cells. Furthermore, we observed an upregulation of ligand-receptor pairs within this niche. These findings suggest a potential association between this cellular arrangement and the underlying mechanisms contributing to SAE or the increased mortality observed in *Anxa1* knockdown mice.

**Supplementary Information:**

The online version contains supplementary material available at 10.1186/s12974-024-03161-0.

## Background

Sepsis, a critical condition marked by life-threatening organ dysfunction resulting from an imbalanced host response to infection, is a significant contributor to global health burdens [1]. In 2017, the Global Burden of Diseases, Injuries, and Risk Factors Study (GBD) documented an alarming estimate of 48.9 million incident cases of sepsis worldwide, with a staggering 11 million sepsis-related deaths [2]. These figures represent approximately 19.7% of all reported global deaths, solidifying sepsis as one of the primary causes of critical illness and mortality. SAE, Sepsis-associated encephalopathy (SAE), a frequent complication in sepsis, manifests through a spectrum of symptoms including alterations in consciousness, ranging from mild cognitive impairment to profound coma, as well as a variety of neurological disturbances such as confusion, cognitive dysfunction, sleep disturbances, hallucinations, and delirium [3–6]. Clinical manifestations of SAE are observed in approximately 70% of septic patients, and this condition has been linked to increased mortality rates. Additionally, survivors often experience long-term neurological impairments. The pathophysiology of SAE is not completely understood, but it is likely multifactorial, involving mechanisms such as oxidative stress, increased cytokines, and a dysregulated host response to infection [7]. Further research is needed to better understand the etiology and pathophysiology of SAE in order to develop more effective prevention and treatment strategies.

The advent of snRNA-seq has revolutionized the field of cell biology, The advent of single-nucleus RNA sequencing (snRNA-seq) has revolutionized the field of cell biology, enabling researchers to unravel the intricate cellular heterogeneity observed in various organs and diseases. In recent years, snRNA-seq has gained significant traction for its ability to dissect the complex landscape of immune cell subsets and uncover distinct cellular signatures associated with a multitude of human diseases [8], including cancers, autoimmune disorders, cardiovascular diseases, and infectious diseases [9–14]. Within the realm of sepsis research, the application of snRNA-seq technology has yielded valuable insights and discoveries [15–18]. Nonetheless, it is paramount to acknowledge that these methods, reliant on tissue dissociation, inherently sacrifice spatial information. Consequently, the precise pathophysiological mechanisms governing the recruitment of inflammatory cells to sites of tissue injury during sepsis remain unknown, as the spatial context is not fully preserved. Spatial transcriptomic platforms offer a powerful means of simultaneously assessing the comprehensive mRNA expression profiles of thousands of genes and overlaying this information onto histological data from adjacent tissue sections. By mapping gene expression back to their original locations, these platforms establish a direct association between histology and gene expression [19]. Integration of snRNA-seq with spatial transcriptomics enhances analytical capabilities and facilitates the in situ-visualization of molecular signatures, resulting in the mapping of a larger repertoire of cell types compared to spatial transcriptomics alone [20]. However, a comprehensive exploration of spatiotemporal omics in the context of sepsis or SAE in the brain remains limited. Therefore, our study aims to employ a combined approach of snRNA-seq and spatial transcriptomics (ST) to map the transcriptional changes occurring in the brains of mice subjected to an endotoxin-induced sepsis model.

Annexin A1 (ANXA1) is a protein that has been extensively studied for its role in various biological processes. In the context of recent studies, ANXA1 is recognized for its anti-inflammatory properties due to its control over leukocyte-mediated immune responses. In the context of viral infections, ANXA1 has a multifaceted role, including promoting apoptosis of granulocytes, efferocytosis of apoptotic cells, and inhibiting the production of pro-inflammatory cytokines [21]. It’s also involved in cellular signaling, hormonal secretion, fetal development, aging, and disease development. Specifically, ANXA1 affects the hypothalamic–pituitary–adrenal (HPA) axis physiology and regulates the blood–brain barrier (BBB) tightness [22]. The multifaceted role of ANXA1 in neuroinflammation has garnered considerable attention in recent literature. The presence of ANXA1 in microglia of aged human brains, as reported by McArthur et al. [23], and its increased expression in Alzheimer’s disease suggest a significant role in the phagocytosis of apoptotic neurons. Zhang et al. [24] further elucidated the neuroprotective effects of ANXA1 tripeptide, highlighting its capacity to inhibit NF-κB p65 and ameliorate neuroinflammation in a rat model of cardiopulmonary bypass. The work of Ding et al. [25] supports these findings, demonstrating that ANXA1 can attenuate neuroinflammation through the FPR2/p38/COX-2 pathway following intracerebral hemorrhage in mice. Recent studies have also shed light on the pathological mechanisms underlying neurodegenerative dementias. Chua et al. [26] observed elevated levels of cleaved ANXA1, indicating a potential failure in the resolution of inflammation and establishing a mechanistic link between ANXA1, amyloid pathology, neuroinflammation, and apoptosis. Moreover, Mao et al. [27] identified SENP6-mediated ANXA1 de-SUMOylation as a contributor to microglial polarization and subsequent neuroinflammation after cerebral ischemia-reperfusion injury. COVID-19 has the potential to induce severe immune reactions, known as cytokine storms [28]. Our previous single-cell sequencing articles on covid-19 patients, anxa1 and its receptor were found to be up-regulated in expression after cytokine storm [29]. Moreover, our recent molecular studies have revealed significant upregulation of ANXA1 in septic encephalopathy. We demonstrated a substantial increase in ANXA1 protein and mRNA levels in a mouse model of septic encephalopathy, with a 2.5-fold and 3-fold elevation respectively at 24 h post-induction. These findings suggest an active involvement of ANXA1 in the central nervous system’s acute phase response to septic challenges.

In this study, we utilized snRNA-seq datasets to spatially map cell types onto transcriptomic anchoring landmarks (spots) within the murine brain. Specifically, we investigated the differentially expressed genes (DEGs) and pathways in the brains of mice subjected to an endotoxin-induced sepsis model. To precisely define the distribution of immune cell transcript expression in these models, we utilized the transcriptomic signatures derived from the Stereo-seq spatial gene expression platform, allowing for the colocalization of glial cells with vascular-associated cells. Through our investigation, we successfully identified key ligand-receptor pairs expressed in glial cells and vascular-associated cells within the brains of mice subjected to an endotoxin-induced sepsis model. These identified pairs play a significant role in facilitating immune cell migration and intercellular communication. By harnessing the high-throughput capabilities of single-nucleus and spatial transcriptome technologies, we have not only advanced our understanding of SAE, but also established a pioneering framework for leveraging spatial information to enhance the interpretation of cellular activation in neuroinflammatory conditions. This approach holds immense potential in deepening our insights into the intricate mechanisms underlying neuroinflammation.

## Materials and methods

### Mice and treatments

C57BL/6 mice were obtained from the Guangdong Medical Laboratory Animal Center, while *Anxa1*^−/−^ mice were procured from GemPharmatech. The mice were housed in the animal care facility at Yuebei People’s Hospital Affiliated to Shantou University Medical College. The mice were maintained under specific conditions, including a regular 12-hour light/dark cycle, a controlled ambient temperature ranging from 23 to 26 °C, and a humidity level maintained between 50 and 60%. The mice used in this study were 8-week-old, which is equivalent to the late adolescence stage in humans (young adult, postnatal day 46–59). Neuroinflammation was induced by a single intraperitoneal injection (IP) of LPS at a dose of 15 mg/kg, obtained from Sigma Aldrich. LPS was diluted in a 0.9% saline solution. The control group, referred to as the Ctrl group in the text, received an injection of normal saline (NaCl). All experimental procedures were conducted in accordance with the guidelines and regulations set by The Medical Animal Care & Welfare Committee of Shantou University Medical College (Approval Number: SUMC2021-332).

### Preparation, sequencing, and alignment for ST libraries

Fresh brain tissues from mice were collected and immediately frozen in OCT compound (Tissue-Tek; Sakura Finetek USA, Torrance, CA, USA). The frozen tissues were sliced transversely into 10 μm thick sections using a CM1950 cryostat (Leica, Wetzlar, Germany). These tissue sections were then affixed to the surface of the Stereo-seq chip. After a 3-minute incubation at 37 °C, the sections were fixed in methanol and further incubated at -20 °C for 40 min. H&E staining was performed on these sections as well as the adjacent tissue sections. Both procedures were imaged using a Ti-7 Eclipse microscope (Nikon, Tokyo, Japan). The libraries were prepared using established methods [30] and sequenced on an MGI DNBSEQ-Tx sequencer. Read 1 contained the coordinate identity (CID) barcode and molecular identifiers (MIDs) (CID: 1–25 bp, MID: 26–35 bp), while read 2 consisted of the cDNA sequences. The raw sequencing reads were filtered and demultiplexed using the SAW pipeline (https://github.com/BGIResearch/SAW). For each library, we generated an expression profile matrix that included the CID information.

### Preparation, sequencing, and alignment of snRNA-seq libraries

The snRNA-seq samples were collected from adjacent frozen sections to those used for Stereo-seq. These sections were carefully transferred into plastic wells on dry ice and subsequently stored at -80 °C refrigerator. Single nucleus suspensions were prepared as previously described [31]. Briefly, mouse brain tissue pieces were homogenized in a Dounce homogenizer with pre-chilled homogenization buffer on ice. The resulting homogenate was filtered through 30 μm MACS SmartStrainers (Miltenyi Biotech, #130-110-915) into a 15 ml conical tube. Subsequently, the tube was centrifuged to pellet nuclei. The pellet was resuspended in blocking buffer and centrifuged again to pellet nuclei. The nuclei were then resuspended in cell resuspension buffer for subsequent snRNA-seq library preparation.

The DNBelab C Series High-throughput Single-Nucleus RNA Library Preparation Kit (MGI, #940-000047-00) was used to construct the sequencing libraries as per the manufacturer’s instructions. In summary, single-nucleus suspensions were employed for droplet generation, emulsion breakage, beads collection, reverse transcription, second-strand synthesis, cDNA amplification, and droplet index products amplification to generate barcodes libraries. The sequencing libraries were quantified using the Qubit™ ssDNA Assay Kit (Thermo Fisher Scientific, #Q10212) and sequenced on the ultra-high-throughput DIPSEQ T1 or DIPSEQ T10 sequencers at the China National GeneBank (Shenzhen, China). Each library had a read length of 41-bp for read 1 and 100-bp for read 2. The raw sequencing reads were filtered and demultiplexed using PISA (v0.2). The reads were then aligned to the reference human genome using STAR (v2.7.4a) and sorted using sambamba (v0.7.0). Finally, a cell versus gene UMI count matrix was generated for each library using PISA.

### SnRNA-seq data analysis

#### Raw data processing

The raw snRNA-seq data were processed using the R package Seurat (v4.1.1) [32]. We merged the gene expression matrix of libraries from each sample, calculated the percentage of reads that mapped to the mitochondrial genome (percent.mt), and counted the number of genes in each cell. The gene number was recorded as ${N}_{express}$ based on the cell with the highest number of genes. Finally, we filtered out cells with less than 400 genes or with more than $0.9\text{*}{N}_{express}$ genes, or with a percent.mt larger than 0.2.

To remove reads originating from ambient RNA, we applied the R package DecontX [33] on each sample with default parameter settings. For the identification and removal of doublets, we used the R package DoubletFinder [34] with default parameters and excluded the top 7.5% of cells that exhibited the closest resemblance to pseudo-doublets.

#### Integration, clustering and annotation of snRNA-seq data

For the integration, clustering, and annotation of snRNA-seq data, we utilized the Seurat package. Firstly, we performed log-normalization using the NormalizeData function, with a scale factor of 10,000. Next, we identified highly variable features using the FindVariableFeatures function, selecting the top 2,000 features. To integrate the data from each dataset, we used the FindIntegrationAnchors and IntegratedData functions. Counts were scaled and centered in the integrated dataset using the ScaleData function. Dimensionality reduction was performed using the RunPCA function, utilizing the first 50 principal components. Subsequently, graph-based clustering was applied using the FindClusters function, with a resolution of 2. Next, we used RunUMAP function to perform Uniform manifold approximation and projection (UMAP) plots. Cluster marker genes were identified using the FindAllMarkers function, and cell types were annotated by comparing the cluster markers with their known canonical markers. Lastly, we merged cell clusters that shared the same markers.

#### Differential gene expression and gene set enrichment analyses of snRNA-seq data

To perform differential gene expression analysis, we employed the FindMarkers function in Seurat. This allowed us to compare gene expression between cells under different conditions, including different cell types (subtypes), time periods, and cells with or without a ligand-receptor (LR). Genes with adjusted *P*-values < 0.05 were considered as differentially expressed genes (DEGs). To perform gene set enrichment analysis, we utilized the enrichGO functions from the R package clusterProfiler (v4.4.4) [35].

#### Calculation of signature score

We initially extracted gene signatures for Astro-1, Astro-2, Micro-1, Micro-2, Neuron, OligoD, OPC, Vas-1, and Cdkn1a^+^ Serpina3n^+^ OligoD from the snRNA-seq data (Supplementary Table 1). To determine the signature score for each cell type, we summed the log-normalized expression values of the corresponding gene signature in each cell. Then, we identified the 99th percentile of the summed value distribution, denoted as x. By dividing the summed value of each cell by x and replacing any values greater than 1 with 1, we obtained the signature score for each cell. Using this approach, we calculated signature scores for all cells across the nine cell types.

### ST data analysis

#### Raw data processing

To precisely capture the gene expression patterns identified by Stereo-seq, we utilized a convolution-based approach to expand the raw spatial expression matrix of single DNB spots into larger pseudo-spots. The coordinates of a segmented-nuclei spot was determined from the single-strand DNA (ssDNA) image of the ST data using CELL BIN Studio (v1.0.6). By merging the DNB spots within each segmented-cells spot, we generated the spatial expression matrix of the pseudo-spots. For each pseudo-spot, its center coordinates were computed as its x-y coordinates.

#### Inferring cell composition in a ST dataset using RCTD algorithm

To determine the cellular composition of each spot, we employed the RCTD algorithm implemented in the spacexr R package (v 2.2.0) [36]. The RCTD algorithm was applied to each dataset using the full model, with our in-house snRNA-seq data serving as the reference. Cell type scores for each spot were normalized using the normalized weights function, and cell type labels were assigned to a spot if the scores surpassed the corresponding threshold value. The threshold values varied depending on the cell type, with values of 0.5 for neurons, 0.25 for Astro-1, 0.2 for OligoD and Vas-1, 0.1 for Astro-2, and 0.05 for OPC, Micro-1, and Micro-2.

#### Inferring cell composition in a ST dataset with cell type signature genes

To identify the cellular composition of a ST dataset using gene signature scores, we initially extracted gene signatures for neurons, Astro-1, OligoD, Vas-1, Astro-2, OPC, Micro-1, and Micro-2 (Supplementary Table 1). We then conducted log-normalization on the ST dataset using the NormalizeData function in Seurat. Using the same approach as with the snRNA-seq data, we calculated the signature score for each cell type in the ST dataset.

#### Integration and clustering of sixteen ST datasets

A total of 20,000 spots were randomly selected from each dataset of ST. These spots were then integrated with Seurat software. To reduce the dimensions of the dataset, we employed RunPCA function with the initial 50 PCs. For graph-based clustering, we used FindClusters function with a resolution of 0.1. Subsequently, we performed UMAP using RunUMAP function.

#### Differential gene expression and gene set enrichment analyses of ST data

We conducted the same differential gene expression and gene set enrichment analyses on the ST data as we did on the snRNA-seq data.

#### Detection of gene co-localization module (Co-locM)

We converted each ST dataset into a pseudo-bulk dataset and performed differential gene expression analysis separately for wild type and *Anxa1*^−/−^ mice (see “Identification of DEGs from bulk and pseudo-bulk data” part in methods for detail). For wild type mice, we compared the pseudo-bulk datasets between 12- or 24-hour and 0- or 72-hour, identifying up-regulated genes with fold change values greater than 2 and *p*-values less than 0.01. The same approach was used for *Anxa1*^−/−^ mice. The up-regulated genes from both sets were then merged to create a candidate gene set. To calculate the co-localization score between two genes (Gene A and Gene B), we analyzed one 12-hour wild type mouse ST dataset. We counted the number of spots (${N}_{spots}$) and extracted the spatial expression matrix. We sorted the spots based on Gene A’s expression and identified the top $0.05*{N}_{spots}$ spots as $\{{A}_{1},{A}_{2}, \dots , {A}_{0.05*{N}_{spots}}\}$. We followed a similar process for Gene B, obtaining the top $0.05*{N}_{spots}$ spots as $\{{B}_{1},{B}_{2}, \dots , {B}_{0.05*{N}_{spots}}\}$. We then determined the overlap between these two sets as ${N}_{A\cap B}$ and the total unique spots as ${N}_{A\cup B}$. The co-location score (${S\_coloc}_{AB}$) was calculated as 0 when ${N}_{A\cap B}$ was less than 50, and as $\frac{{N}_{A\cap B}}{{N}_{A\cup B}}$ when ${N}_{A\cap B}$ was 50 or greater. This calculation was repeated for all genes in the candidate gene set. Using hierarchical clustering analysis, we identified a gene module consisting of 130 genes based on the co-localization scores. This module was named Co-locM (Supplementary Table 6). We visualized the Co-locM by calculating the co-localization scores of the candidate gene set in all sixteen ST datasets.

#### Grouping spots based on cell type label combinations

From a 12-hour wild type mouse ST dataset, we gathered spots with various labels such as Astro-1, Astro-2, Micro-1, Micro-2, Neuron, OligoD, OPC, Vas-1, or Cdkn1a^+^ Serpina3n^+^ OligoD. These spots were then categorized into different groups based on their label combinations. We performed a count of spots within each group and specifically examined spot groups containing more than 100 spots. Following the same approach, we identified spot groups in eight ST datasets from both wild type and *Anxa1*^−/−^ mice at 12- and 24-hour time points. We objective was to identify spot groups that consistently appeared in all eight datasets. As a result, we identified a total of 46 spot groups across these datasets.

#### Calculation of module score

To calculate the module score for Co-locM, we employed the same method that we used for calculating the signature score.

#### Identification of ligand-receptor pairs specifically activated in wild type or Anxa1^***−/−***^ mice at 12- and 24-hour time points

We extracted a total of 2,033 literature-supported ligand-receptor pairs (LRs) specific to mice from the CellTalkDB database [37]. To calculate the activity of a LRs in a 12-hour wild type mouse ST dataset, we followed a specific procedure. Firstly, we calculated the co-localization score (${S\_coloc}_{LR}$) between the ligand and the receptor, using the same method described in the “Detection of gene co-localization module (Co-locM)” section of our methods. Next, we randomly selected 10,000 gene pairs from the ST dataset, ensuring that the two genes in each pair were not identical. We then calculated the co-location scores for these randomly selected gene pairs, resulting in a random score set denoted as $\{{S\_coloc}_{1},{S\_coloc}_{2}, \dots , {S\_coloc}_{\text{10,000}}\}$. The frequency at which $\{{S\_coloc}_{1},{S\_coloc}_{2}, \dots , {S\_coloc}_{\text{10,000}}\}\ge {S\_coloc}_{LR}$ was determined as ${freq}_{LR}$. Finally, the LR activity was calculated as $-log2\left({freq}_{LR}\right)$. In the same manner, we calculated the activity of all 2,033 LRs across the sixteen ST datasets obtained from wild type and *Anxa1*^−/−^ mice at different time points following LPS treatment. We identified LRs that were specifically activated in wild type mice at 12- and 24-hour time points. To do this, we grouped the LR activity values obtained from 12- and 24-hour wild type mice ST datasets as Set1, and the values from the other two time points as Set2. We replaced values larger than $-log2\left(0.05\right)$ with 1 and other values with 0 in both sets. We then used Wilcox rank sum test to compare the two sets. If Set1 had a higher rank than Set2, and the *P*-value was less than 0.05, we considered the LR as specifically activated in wild type mice at 12- and 24-hour time points. We applied this method to all LRs to identify those that were specifically activated in either wild type or *Anxa1*^−/−^ mice at 12- and 24-hour time points.

### Bulk and pseudo-bulk gene expression data analysis

#### Data acquisition and processing

We retrieved bulk RNA-seq and microarray data, along with their corresponding sample information, for mouse brain tissue under various conditions from the GEO database (https://www.ncbi.nlm.nih.gov/geo/). The datasets we used were identified with the accession IDs GSE153369, GSE7814, GSE6019, GSE53874, GSE205140, and GSE9524. We downloaded and utilized the processed and normalized matrices from these datasets, specifically selecting relevant samples for further analysis.

To generate the pseudo-bulk RNA-seq dataset for each ST dataset, we summed the expression matrix of spots based on genes. We then calculated the rpm (reads per million mapped reads) value for each gene, which was utilized for subsequent analysis.

#### Identification of DEGs from bulk and pseudo-bulk data

We identified DEGs between two groups of mice using both bulk and pseudo-bulk data. For example, we compared the gene expression levels between different time periods following LPS treatment and between wild type and *Anxa1*^−/−^ mice. We calculated the fold change values (between the two groups) and *P*-values. Specifically, we conducted *t*-tests when there were more than two samples in each group, enabling us to ascertain significant differences.

#### Western blotting

Brains of mouse were dissected and homogenized using tissue grinders (Kimble) in 1.5 ml tubes. The homogenization process involved the use of lysis buffer containing protease inhibitors (Complete Mini; Roche). After 1 min of homogenization, cellular debris was removed by centrifugation at 12,000×g for 10 min at 4 °C. The resulting supernatant was collected and subjected to denaturation at 95 °C for 10 min. Tissue lysates were separated using SDS-PAGE (Bio-Rad) and transferred to a polyvinylidene fluoride membrane. To prevent non-specific binding, the membranes were blocked for 1 h using 5% non-fat dry milk in phosphate buffered saline (PBS) containing 0.5% Tween-20 (PBST). Subsequently, the membranes were probed with primary antibodies against *Anxa1* (NR1, Abcam, ab109182) and glyceraldehyde 3-phosphate dehydrogenase (GAPDH, Beyotime, AF0006), which served as a loading control. After three washes within PBST, a HRP-labeled secondary antibody (CWS) was applied at room temperature (RT) for 1 h using a dilution buffer for secondary antibodies (Beyotime). Subsequently, the membranes were washed three more times with PBST. The bands were visualized using the Immobilon Western ECL system (Millipore) and analyzed using ImageJ Analysis software.

For real-time Polymerase Chain Reaction (PCR), we added 1 μL of template DNA and 1 μL of primer mixture to the 2x Taq Plus Master Mix II (Vazyme, P213). Ultrapure water was then added to achieve a final reaction volume of 25 μL. The reaction mixture underwent an initial denaturation at 95 °C for 5 min, followed by 32 cycles of denaturation at 95 °C for 30 s, annealing at 55 °C for 30 s, and extension at 72 °C for 30 s. Eventually, there was a final extension at 72 °C for 10 min. After the reaction, 7 μL of the sample was electrophoresed on a 1.5% agarose gel containing 0.01% ethidium bromide at 120 V for 35 min. The gel was then subjected to UV imaging and subsequent analysis.

#### Immunofluorescence

Mice were anesthetized using an intraperitoneal injection of 20% urethane (0.01 ml/g) and subsequently perfused through the heart with 0.01 M PBS (pH 7.4), followed by 4% paraformaldehyde (PFA). After perfusion, the brains were dissected and post-fixed in 4% PFA overnight. To protect the tissue during freezing, they were then immersed in a 30% sucrose solution at 4 °C. Serial coronal sections, with a thickness of 20 μm, were obtained using a Leica CM3050S. The slides were warmed to RT and washed three times with PBS. To enhance permeability of the tissue, the sections were washed with 0.3% Triton X-100 in PBS for 10 min. Non-specific binding was blocked by incubating the tissue sections with QuickBlock™ Blocking Buffer (P0260) at RT for 10 min. The sections were then incubated overnight at 4 °C with rabbit anti-Iba1 (Wako, 019-19741, 1:1000), CD68 (Abcam, ab53444, 1:250), and anti-GFAP (CST, #3670, 1:1000) antibodies in Primary Antibody Dilution Buffer (P0103). After three washes with PBS, a secondary antibody (Invitrogen) was applied and incubated at RT for 1 h. Afterward, the sections were washed three more times with PBS for 10 min. The sections were then washed and mounted using ProLong™ Gold Antifade Mountant (P36930) and imaged using a fluorescence microscope.

## Results

### snRNA-seq analysis of the mouse brain over the course of lipopolysaccharide challenge

This study employed both wild type and *Anxa1* knockout (*Anxa1*^*−/−*^) C57BL/6 mice as experimental models. To validate the successful generation of the *Anxa1*^*−/−*^ mouse model, Western blotting was performed on brain tissue samples obtained from both wild type and *Anxa1*^*−/−*^ mice. As anticipated, the ANXA1 protein band was not detected in the *Anxa1*^*−/−*^ mice, while it was observed in the wild type mice, thereby confirming the successful construction of *Anxa1*^*−/−*^ mouse model. Furthermore, Real-time Polymerase Chain Reaction (PCR) was utilized to validate the absence of *Anxa1* in the tails of the mice, providing further support to the findings obtained through Western blot analysis. This additional analysis further strengthens the evidence and confirms the successful establishment of the *Anxa1*^*−/−*^ model (Supplementary Fig. 1C). Both wild type and *Anxa1*^*−/−*^ mice were intraperitoneally injected with either *Escherichia coli* lipopolysaccharide (LPS; serotype 0111: B4, Sigma; 15 mg/kg body weight) or a control solution of endotoxin-free saline. During the 132-hour observation period, notable weight loss was observed in both wild type and *Anxa1*^*−/−*^ mice following LPS administration. However, after 72 h, their weights stabilized or even increased, suggesting the occurrence of intense inflammatory responses during the initial phases of simulated endotoxemia (Supplementary Fig. 1D).

**Fig. 1:**
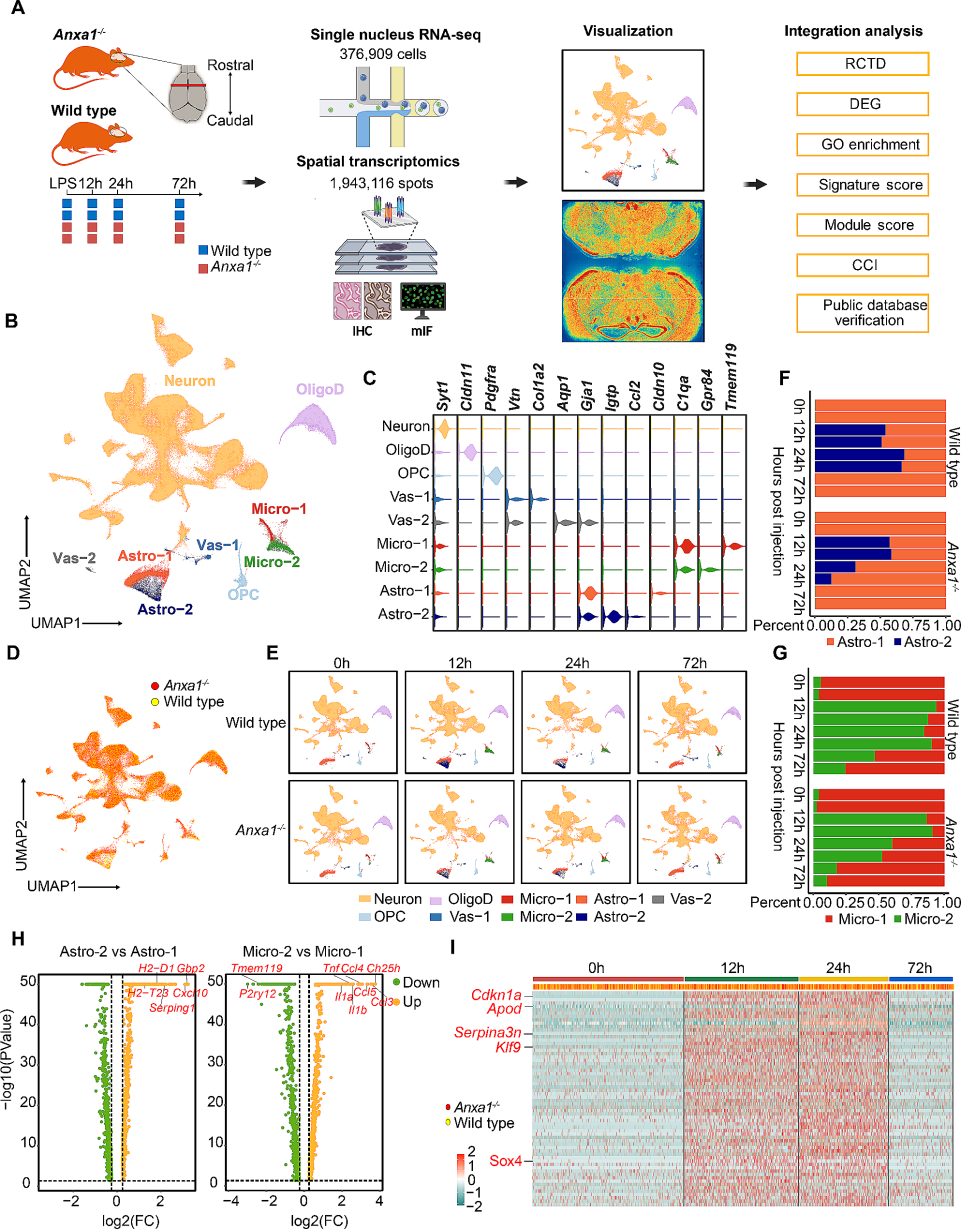
Single-nucleus transcriptomic profiling of the mouse brain following peripheral LPS stimulation. (**A**) Schematic diagram illustrating the acquisition and analysis workflow of the spatial transcriptomics (ST) maps. Brain tissue samples from 8 wide type and 8 *Anxa1* knockout C57BL6 mice were subjected to Stereo-seq, resulting in a total of 1,943,116 spots. Paired brain tissue samples of the wild type and *Anxa1*^*−/−*^ mice were also subjected to snRNA-seq, and 376,909 qualified nuclei were identified. The processed data was subjected to dimensionality reduction using Uniform Manifold Approximation and Projection (UMAP) and spatial cell types mapping for visualization, respectively. Finally, a combined analysis of the single-nucleus sequencing results and the spatial transcriptome sequencing results was performed. (**B**) UMAP plot of the snRNA-seq data. Each cluster in the plot corresponds to a specific cell type and is color-coded accordingly. The cell types included in the plot are Oligodendrocytes (OligoD), Vascular cells (Vas), Microglial cells (Microglia), Astrocytes (Astro), and Oligodendrocyte precursor cells (OPC). (**C**) Expression of canonical markers in each cell type cluster. (**D**) The UMAP plot displays cells from both the wild type and *Anxa1*^−/−^ mice, with each cell color-coded based on the genotype of the respective mouse. (**E**) The UMAP plots demonstrate the changes in cell dynamics after administering LPS to both wild type and *Anxa1*^−/−^ mice. (**F**-**G**) The relative proportions of Astro-1 and Astro-2 cells (**F**), as well as Micro-1 and Micro-2 cells (**G**), were compared at different time intervals following LPS treatment in both wild type and *Anxa1*^−/−^ mice. (**H**) Volcano plots were generated to visualize the differentially expressed genes (DEGs) between Astro-2 and Astro-1 cells, as well as between Micro-2 and Micro-1 cells. The X-axis and Y-axis represent the log_2_ fold-change differences between the compared cell types and the statistical significance as the negative log_10_ of DEG *P*-values, respectively. Significantly up-regulated and down-regulated genes are indicated by orange and green dots, respectively, while non-significant genes are represented as black dots. (**I**) Heatmap displaying the DEGs between two groups of Oligodendrocytes. The first group consists of Oligodendrocytes from wild type and *Anxa1*^−/−^ mice at 12- or 24-hour, while the second group comprises Oligodendrocytes from wild type and *Anxa1*^−/−^ mice at 0- or 72-hour. Only the up-regulated genes with a log2 fold-change value greater than 1 and an adjusted *p*-value smaller than 0.05 are shown. Oligodendrocytes from wild type mice are colored in yellow, while those from *Anxa1*^*−/−*^ mice are colored in red

We employed both wild type and *Anxa1*^*−/−*^ C57BL/6 mice as experimental models to investigate the intricate cellular composition and transcriptional pathways in the central nervous system (CNS) during sepsis or SAE. Through snRNA-seq and high-resolution spatial transcriptomic analyses, we examined brain tissues obtained at 12 h, 24 h, and 72 h post LPS challenge. After rigorous quality control and filtering procedures, a toral of 376,909 high-quality single nucleus cells were obtained. On average, each cell contained approximately 3028 unique molecular identifiers (UMIs) and expressed 1,431 genes (Supplementary Table 2). Through our rigorous analysis, we successfully identified nine clusters representing six distinct cell types within the CNS. These cell types encompassed neurons, microglia, astrocytes, oligodendrocytes, oligodendrocyte precursor cells, and vascular cells (Fig. 1B). Specifically, neurons were characterized by the expression of *Syt1* [38], oligodendrocytes exhibited the marker *Cldn1* [39], and oligodendrocyte precursor cells displayed *Pdgfra* expression [40–42]. Notably, microglia and astrocytes were further subdivided into two subclusters based on their distinctive gene expression patterns. Microglia were classified as *Tmem119*^*+*^ (Micro-1) [43, 44] and *Gpr84*^*+*^ (Micro-2) [45], while astrocytes were divided into *Cldn10*^*+*^ (Astro-1) and *Igtp*^*+*^ (Astro-2) subclusters [46]. Furthermore, vascular-associated cells were also classified into two subclusters: *Col1a2*^*+*^ (Vas-1) [47] and *Vtn*^*+*^ (Vas-2) [48] (Fig. 1B and C). Our findings are consistent with previous studies investigating cellular diversity in the mouse cortex and hippocampus [49]. Interestingly, no substantial differences in cell types were observed between *Anxa1*^*−/−*^ and wild type mice following LPS challenge, suggesting that *Anxa1* does not influence the composition of cells involved in brain inflammatory responses (Fig. 1D). However, notable changes were detected in the UMAP plots (Fig. 1E), particularly in the Astro-2 and Micro-2 populations. These subpopulations initially exhibited an increase in abundance, followed by a subsequent decrease at 24 h, and eventually returned to baseline levels by 72 h in both mouse groups. At the 12-hour time point, we observed a comparable increase in the astro-2 and micro-2 populations in both *Anxa1*^*−/−*^ and wild type mice. This initial response suggests a shared early reaction in these populations irrespective of the *Anxa1* status. However, the subsequent responses diverged significantly between the two groups. In wild type mice, the astro-2 and micro-2 populations reached a stable state at 24 h, indicating a controlled and self-regulatory response in the presence of *Anxa1*. Contrastingly, *Anxa1*^*−/−*^ mice exhibited a rapid decline in both astro-2 and micro-2 populations during this same time point. This decline underscores the potential critical role o*f Anxa1* in sustaining these cell populations beyond the initial response phase (Fig. 1F-G). We conducted a thorough analysis of differentially expressed genes (DEGs) between Astro-2 and Astro-1 subpopulations, which uncovered significant upregulation of genes such as *Gbp2*, *Cxcl10*, *Serping1*, and MHC-associated genes *H2-D1* and *H2-T23* in Astro-2 (Supplementary Table 3). In contrast, a comparison with Micro-1 revealed the downregulation of steady-state genes *Tmem119* and *P2ry12*, alongside upregulation of inflammatory genes including *TNF*, *Il1a*, *Il1*, and chemokines *Ccl3*, *Ccl4*, and *Ccl5* in Micro-2 (Supplementary Table 4). These alterations in the Micro-2 subpopulation align with the well-known characteristics of microglia activated by LPS, as previously reported [50]. Furthermore, pathway analysis highlighted that the upregulated genes in both Astro-2 and Micro-2 were primarily enriched in pathways associated with viral response, biotic stimulus regulation, and innate immune response (Supplementary Fig. 1E). Moreover, to investigate the transcriptional impact of LPS stimulation on oligodendrocytes as a whole, we also conducted a differential expression analysis on a cluster of oligodendrocytes previously identified. This analysis revealed dynamic changes in their transcriptional profiles. For instance, we found that oligodendrocytes exhibit distinct gene expression patterns at different time points, with genes showing high expression at 12 h and 24 h, while displaying deceased expression or no expression at 0 h and 72 h following the challenge (Fig. 1I). Within this set of genes, we identified *Serpina3n* and *Cdkn1a*, which have established associations with specific diseases and differentiation processes. In addition, genes such as *Sox4*, *Apod*, and *Klf9* were also observed. These findings suggest that oligodendrocytes undergo distinct differentiation states at 12 h and 24 h compared to other time points. These particular oligodendrocytes, referred to as *Cdkn1a*^*+*^*Serpina3n*^*+*^ OligoD, exhibited enriched pathways related to cytoplasmic translation, macromolecule biosynthetic process regulation, and viral response (Supplementary Figure S1F). Our snRNA-seq results indicate a significant increase in the proportions of Astro-2, Micro-2, and *Cdkn1a*^*+*^*Serpina3n*^*+*^ OligoD within 24 h of LPS challenge, emphasizing their pivotal roles during the initial phases of simulated endotoxemia.

### Spatially-resolved transcriptomics of the mouse brain over the course of lipopolysaccharide challenge

Spatial information is critical for understanding cell-cell interactions that occur within tissues. Regrettably, this valuable information is absent from the snRNA-seq data. To gain deeper insights into the specific cell type that exerts the most significant impact on the inflammatory response at 12 h and 24 h, as well as to investigate the spatial relationships and potential mechanisms of interaction among these cell types, we employed Stereo-seq technology. This advanced method allowed us to capture in situ gene expression profiles. The Stereo-seq chips used were designed with capture probes incorporating a 25 bp coordinate identity barcode, a 10 bp molecular identity barcode, and a 22 bp poly T tail to facilitate mRNA hybridization. Brain tissues were collected from both wild-type and *Anxa1*^*−/−*^ mice at 12 h, 24 h, and 72 h following LPS challenge. These tissues were subsequently embedded in optimal cutting temperature (OCT) compound. Utilizing this approach, we prepared serial cryosections with a thickness of 10 μm for Stereo-seq, hematoxylin and eosin (H&E) staining, and immunohistochemical (IHC) staining. Following the initial snRNA-seq analysis performed on 16 samples (including wild type and *Anxa1*^*−/−*^ at 0 h, 12 h, 24 h, and 72 h, with two samples at each time point), we proceeded to conduct spatial transcriptome sequencing. The Stereo-seq chips utilized in this study were designed with capture spots measuring 220 nm in diameter and spaced at a distance of 500 nm from each other. To generate a comprehensive expression matrix with single-nucleus cell resolution, we employed a registration process that aligned the ssDNA image with the DNB image. We then utilized the StereoCell package for precise cell segmentation, thereby enabling the creation of a spatial gene expression matrix. To ensure the accuracy and reliability of gene annotation, we established stringent criteria of a minimum of 660 gene types and 1,300 counts per cell bin across all utilized chips. Notably, the gene capture and UMI numbers demonstrated a consistent pattern across samples obtained from both wild type and *Anxa1*^*−/−*^ mice. This observation serves as a strong indicator of the acquisition of high-quality spatial transcriptomic data (Supplementary Figures S2A and S2B). To conduct cell annotation, we generated distinct marker gene sets for each cell cluster by utilizing dimensionality-reduced snRNA-seq data. These gene sets were further visualized, demonstrating their remarkable specificity (Supplementary Figure S2C). To ensure comprehensive analysis, we examined 20,000 spots per sample for UMAP graph visualization, successfully mitigating any potential batch effects (Supplementary Figures S2D and S2E). Additionally, we devised a marker gene signature score for each cell cluster using spatial transcriptomics data. The accuracy of this annotation was confirmed through robust cell type decomposition (RCTD) [51] (Supplementary Figures S2F and S2G). Furthermore, by comparing the RCTD scores with the marker gene signature scores, we established the consistency and validity of our annotation outcomes.

**Fig. 2:**
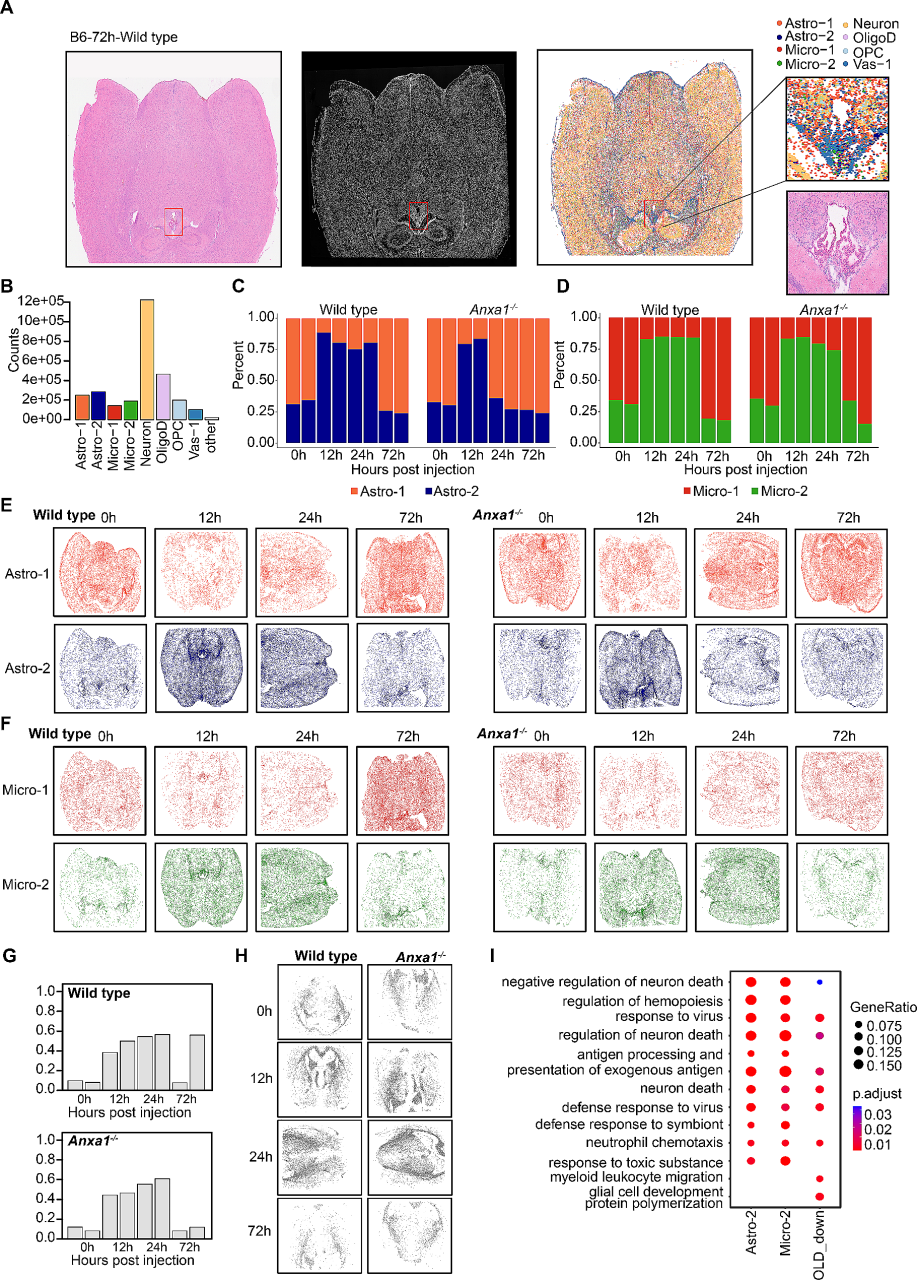
Spatially-resolved transcriptomic analysis following peripheral LPS stimulation. (**A**) Hematoxylin and eosin (H&E) staining, single-stranded DNA (ssDNA) picture, and cell type mapping of the ST slide from a wild type mouse at 72-hour. The spatial expression signal in the slide was detected using Stereo-seq technology, and the raw spatial expression matrix was transformed into spots based on the ssDNA picture. The cell types within the spots were then identified using the robust cell type decomposition (RCTD) method. In the right panel, the H&E staining and cell type labels around the third ventricle are emphasized. (**B**) The number of spots in the spatial transcriptome (ST) data with the eight assigned cell type labels. Spots that are not categorized into any of the eight cell type labels, namely Astro-1, Astro-2, Micro-1, Micro-2, Neuron, OligoD, OPC, and Vas-1, are labeled as “other”. (**C**-**D**) The relative proportions of Astro-1 and Astro-2 spots (**C**), as well as Micro-1 and Micro-2 spots (**D**), were compared at different time intervals following LPS treatment in both wild type and *Anxa1*^−/−^ mice. (**E**-**F**) Spatial map of Astro-1 or Astro-2 spots (**E**), as well as Micro-1 or Micro-2 spots (**F**) at different time intervals following LPS treatment in both wild type and *Anxa1*^−/−^ mice. (**G**) Proportions of Cdkn1a^+^ Serpina3n^+^ OligoD spots relative to all Oligodendrocyte spots across the ST data. (**H**) Spatial map of Cdkn1a^+^ Serpina3n^+^ OligoD spots across the ST data. (**I**) The enriched GO BP-terms for the DEGs between Astro-2 and Astro-1, Micro-2 and Micro-1, as well as Cdkn1a^+^ Serpina3n^+^ OligoD and OligoD in the ST data. The top six GO BP-terms for the up-regulated genes in each category-Astro-2, Micro-2, and Cdkn1a^+^ Serpina3n^+^ OligoD are presented

H&E staining unveiled a considerable population of ependymal cells within the third ventricle, encompassed by astrocytes, aligning with spatial transcriptomic annotations. Spatially resolved transcriptomic analysis revealed the distribution of neurons, astrocytes, and microglia throughout the entire brain slice, with neurons exhibiting the highest prevalence, in accordance with previous research (Fig. 2A) [52]. We conducted a thorough analysis of the cellular clusters within the spatial transcriptome data to ascertain their respective proportions, and observed no notable disparities in terms of cell type composition or quantities between wild type and *Anxa1*^*−/−*^ mice. Neurons constituted the largest proportion, followed by glial cells, with vascular cells being the least abundant (Fig. 2B and Supplementary Figure S2H). We examined the ratios of Astro-2 and Micro-2 subtypes at different time points, indicating a noteworthy upregulation of both subtypes in both mouse brains at 12 h following LPS challenge. Nevertheless, while Micro-2 levels continued to be significantly elevated at 24 h in both groups, Astro-2 levels remained sustained in the wild type mice but reverted to baseline in the *Anxa1*^*−/−*^ mice. This observed pattern was consistently supported by the results obtained from the snRNA-seq analysis. At the 72-hour mark following the challenge, both Astro-2 and Micro-2 levels reverted to their baseline levels (Fig. 2C and D). To validate and ascertain the observed patterns during simulated endotoxemia, we employed spatial visualization to analyze the temporal trends of the inflammation-related cell clusters (Fig. 2E and F). Furthermore, for the analysis of oligodendrocytes, we used a specific gene set, *Cdkn1a*^*+*^*Serpina3n*^*+*^ OligoD, derived from the snRNA-seq data, which demonstrated a high degree of specificity (Supplementary Figure S2J). Gene module scores were assessed in the spatial transcriptome data, utilizing a predefined threshold of 0.4 for the specific gene set. Clusters surpassing this threshold were identified as *Cdkn1a*^*+*^*Serpina3n*^*+*^ OligoD (Supplementary Figure S2K). Within a 24-hour timeframe, this particular population exhibited a significantly increase, amounting to approximately 50% in both mouse brains, thereby corroborating the findings obtained through snRNA-seq analysis. Moreover, the spatial visualization of these oligodendrocytes consistently demonstrated similar trends (Fig. 2G and H). The analysis of the spatial transcriptome data further confirmed that alterations in cell proportions of Astro-2, Micro-2, and *Cdkn1a*^*+*^*Serpina3n*^*+*^ OligoD were in concordance with those observed in snRNA-seq analysis. The Gene Ontology (GO) enrichment analysis revealed that these clusters were primarily associated with pathways such as “response to virus”, “regulation of neuron death” and “defense response to virus”, which are all pertinent to neuron death and defense responses, particularly evident 24 h following LPS stimulation. In contrast, Astro-2 and Micro-2 subtypes were implicated in additional pathways related to the regulation of neuron death and inflammatory responses, including “neutrophil chemotaxis” and “myeloid leukocyte migration” (Fig. 2I). Furthermore, the spatial transcriptome gene expression mapping revealed that while a significant proportion of spots in both mouse strains comprised a single nucleus cell type, there were also a notable number of spots containing multiple cell types (Supplementary Figure S2I).

The integrated findings from our snRNA-seq and spatial transcriptomics analyses demonstrate a significant upregulation of Astro-2, Micro-2, and *Cdkn1a*^*+*^*Serpina3n*^*+*^ OligoD in both wild-type and *Anxa1*^*−/−*^ mice at 12 h following LPS challenge. By the 24-hour timepoint, Micro-2 and *Cdkn1a*^+^*Serpina3n*^+^ OligoD remain elevated, while Astro-2 exhibits upregulation in wild-type mice but returns to baseline levels in *Anxa1*^*−/−*^ mice. The spatial visualization of the transcriptome data further confirms that within 24 h post-challenge, the proportions of these three cell types, namely Astro-2, Micro-2, and *Cdkn1a*^+^*Serpina3n*^+^ OligoD, experience a significant increase, thereby playing key roles in neuron death and defense response pathways in both experimental groups.

### snRNA-seq combined spatial transcriptome analysis reveals special pathological niche in mouse brain during sepsis

**Fig. 3:**
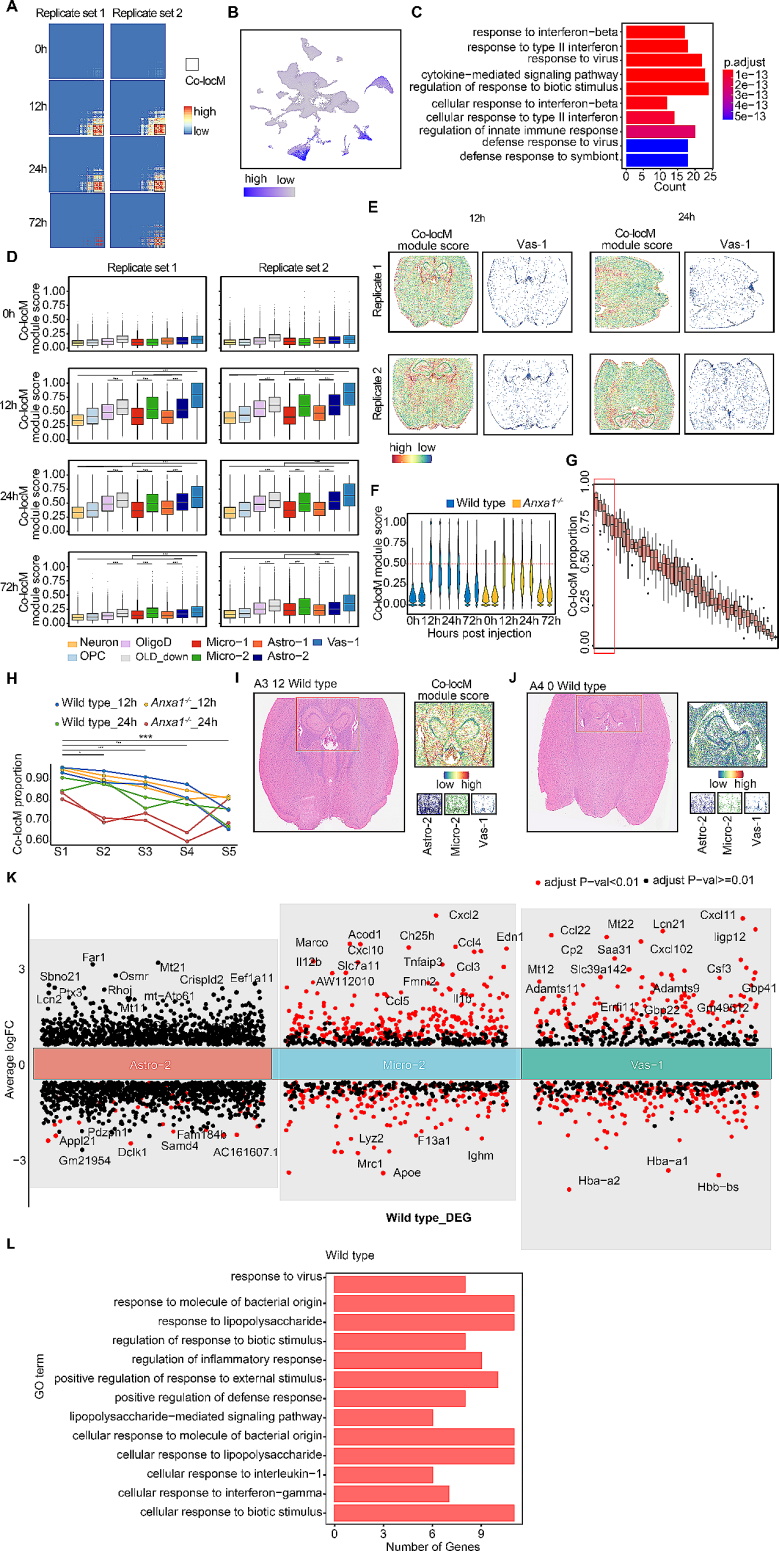
Diversity and spatial distribution of microglia, astrocytes, and Oligodendrocytes following peripheral LPS stimulation. (**A**) Heatmap illustrating the gene co-localization module (Co-locM) in the ST data of wild type mice from different time periods after LPS treatment. (**B**) Visualization of Co-locM module score in the UMAP plot of the snRNA-seq data. (**C**) The enriched GO BP-terms in the genes of the Co-locM module. The top ten GO BP-terms are shown. (**D**) Distribution of the Co-locM module score in spots assigned with different cell type labels in the ST data of wild type mice from different time periods. The boxplot for each spot type is color-coded based on its label type. T tests (unpaired samples, two-tailed) were conducted between different spot types, and the *P*-value or the maximum *P*-value of a test set was reported. Significance levels are indicated as follows: *** *P* < 0.001. (**E**) Spatial distribution of the Co-locM module score and the spatial map of Vas-1 in the ST data obtained from wild type mice at 12- and 24-hour time points. (**F**) Distribution of the Co-locM module score in all spots within the ST data. The ST data is color-coded based on the mice genotype. Spots with a score above 0.5 were assigned the Co-locM label. (**G**) Distribution of Co-locM spot proportion across 46 spot groups. The spots were selected based on the presence of any of the nine labels (Astro-1, Astro-2, Micro-1, Micro-2, Neuron, OligoD, OPC, Vas-1, and Cdkn1a^+^ Serpina3n^+^ OligoD) and grouped according to the combination of labels present in each ST dataset. The Co-locM spot proportion in each spot group was calculated and plotted across eight ST datasets from mice at 12- and 24-hour time points. The top five spot types with the highest Co-locM spot proportion are highlighted. (**H**) Distribution of Co-locM spot proportion in the top five spot groups. Spot Group 1 (S1) consists of spots labeled with Micro-2, Astro-2, and Vas-1; Spot Group 2 (S2) comprises spots labeled with OligoD, Micro-2, Astro-2, and Cdkn1a^+^ Serpina3n^+^ OligoD; Spot Group 3 (S3) includes spots labeled with Astro-2 and Vas-1; Spot Group 4 (S4) consists of spots labeled with OligoD, Astro-2, and Cdkn1a^+^ Serpina3n^+^ OligoD; Spot Group 5 (S5) encompasses spots labeled with Micro-2 and Vas-1. The Co-locM proportion between S1 and S2, S3, S4, and S5 is compared using a T-test (paired samples, one-tailed). Significance levels are indicated as follows: *P < 0.05, ***P < 0.001. (**I**-**J**) Spatial maps of Astro.2, Micro-2, and Vas-1 spots in twFo different regions from wild type mice. The first region, as a Co-locM hot spot (**I**), is from a 12-hour mouse and represents a region where all three cell types are co-localized. The second region is a paired region from a 0-hour mouse (**J**). The maps illustrate the distribution and co-localization of these three cell types in each region. (**K**) DEG analysis showing up- and down-regulated genes across Astro-2, Micro-2 and Vas-1 clusters in snRNA-seq data of wild type mice. An adjusted *P* value < 0.01 is indicated in red, while an adjusted *P* value ≥ 0.01 is indicated in black. DEG, differentially expressed genes. (**L**) The GO terms obtained by performing GO enrichment analysis on the top 30 genes of Astro-2, Micro-2 and Vas-1 clusters based on the log_2_ FC value in the snRNA-seq data of wild type mice

To delve deeper into the spatial distribution of inflammatory responses, we conducted a meticulous spatial co-localization analysis of 682 up-regulated genes at 12 h and 24 h after LPS challenge, using the spatial transcriptomic data. This comprehensive analysis revealed a specialized gene module consisting of 130 genes, which demonstrated consistent expression patterns in both wild-type and *Anxa1*^*−/−*^ mice (Fig. 3A and Supplementary Figure S3A). Analysis of gene expression profiles in brain slices collected at 0 h, 12 h, 24 h, and 72 h after the challenge substantiated the augmented expression of this particular gene module at the 12-hour and 24-hour time points (Supplementary Figure S3B). Furthermore, the snRNA-seq data revealed that these genes exhibited a high level of expression in the Astro-2 and Micro-2 clusters, further reinforcing their involvement in mediating inflammatory responses at the spatial transcriptomic level. Importantly, these findings indicate that there were no significant transcriptional changes observed in the other cell clusters following LPS stimulation (Fig. 3B). GO analysis provided valuable insights into the functional properties of this gene module, indicating its involvement in crucial inflammatory pathways including “response to interferon-beta”, “response to type II interferon”, “response to virus”, and “cytokine-mediated signaling pathway”. These findings suggest the regulatory role of this module in inflammatory responses within the mouse brain, particularly 24 h after the stimulation (Fig. 3C). Subsequent analysis aimed to examine the distribution patterns of this module across different cell types within brain tissue slices. Notably, Astro-2, Micro-2, and *Cdkn1a*^+^*Serpina3n*^+^ OligoD demonstrated elevated module scores compared to non-inflammatory subpopulations at both the 12-hour and 24-hour time points following the stimulation. Significantly, the Vas-1 cluster showed pronounced upregulation and displayed the highest scores within the identified gene module (Fig. 3D). Spatial visualization analysis conducted at 12 h and 24 h post stimulation substantiated these findings, illustrating that the regions with the highest scores were primarily localized around blood vessels. This observation implies a vascular-centric distribution pattern of this module within the brain tissue (Fig. 3E). A parallel trend was observed in *Anxa1*^*−/−*^ mice, providing evidence that *Anxa1* plays a negligible role in the regulation of inflammatory responses within these specific cell types during endotoxemia simulation (Supplementary Figure S3C and Figure S3D). In summary, our integrated analysis of snRNA-seq and Stereo-seq revealed that identified gene module showcased heightened expression not only in the Astro-2 and Micro-2 clusters but also manifested augmented scores within the Vas-1 cell cluster. These findings collectively indicate a notable inflammatory activity within these regions in both wild type and *Anxa1*^*−/−*^ mice at the 12-hour and 24-hour time points following LPS stimulation.

Given the existence of diverse cell types within certain spatial transcriptome spots (Supplementary Figure S2I), it became imperative to explore the precise distribution pattern of a 130-gene module within Vas-1 cell spots. To this end, we implemented a threshold value of 0.5, wherein any spot score surpassing this threshold denoted the presence of expression for the forementioned 130 genes (Fig. 3F). To substantiate the distribution pattern within spatial transcriptome spots comprising multiple cell types, a comprehensive analysis of the data was undertaken. Notably, it was observed that the gene module under investigation exhibited pronounced enrichment in a specific set of 46 mixed cell type spots (Fig. 3G). Furthermore, the application of paired t-tests provided compelling evidence of the module’s substantial enrichment (*P* < 0.05) in spots containing mixed Astro-2/Micro-2/Vas-1 cells, particularly in brain slices derived from both wild type and *Anxa1*^*−/−*^ mice, at 12 h and 24 h subsequent to LPS stimulation (Fig. 3H). To facilitate spatial visualization, our attention was directed towards the hippocampus, lateral ventricle, and third ventricle regions. Intriguingly, a closer examination revealed a higher concentration of Astro-2 and Micro-2 cells in the vicinity of blood vessels at the 12-hour mark following stimulation in wild type mice, which aligns consistently with previous findings (Fig. 3I and J). Furthermore, an in-depth analysis of the snRNA-seq data pertaining to the genes upregulated in Astro-2, Micro-2, and Vas-1 cell types (Fig. 3K) was followed by a subsequent GO enrichment analysis. Remarkably, the results of this analysis indicated notable associations with “viral response” and “response to biotic stimulus” (Fig. 3L). The observed enrichments serve to validate the findings of the Co-locM module obtained from spatial transcriptomic analysis, thereby highlighting the coherence and agreement between different data modalities. Notably, the examination of brain slices derived from *Anxa1*^*−/−*^ mice exhibited analogous patterns of cell distribution surrounding Vas-1 cells at both 12-hour and 24-hour intervals post LPS stimulation, with no significant differences compared to wild type mice group. This suggests that the knockout of *Anxa1* did not induce any alterations in the distribution of inflammatory cells around Vas-1 cells during the simulated sepsis condition (Supplementary Figures S3E, S3F). Moreover, a subsequent GO enrichment analysis conducted on the snRNA-seq data further reinforces these findings by reaffirming the involvement of these genes in the “viral response” process, in accordance with the spatial transcriptomic analysis (Supplementary Figures S3G, S3H).

By employing co-localization analysis, differential expression analysis and GO enrichment analysis, we were able to discern that a module comprising 130 genes primarily associated with inflammation response exhibited heightened expression in Astro-2, Micro-2 and Vas-1 subtypes (specifically, the V1A2M2 colocalization structure) at both 12-hour and 24-hour time points subsequent to LPS stimulation, in both the wild type and *Anxa1*^*−/−*^ mouse brain. Intriguingly, an exploration of the 130-gene module across various published databases revealed its presence in mouse models infected with parasites or viruses, while no occurrence was observed in models stimulated with LPS. This indicates a potential protective role of the V1A2M2 complex in response to infection scenarios (Supplementary Figure S6).

### Cell-cell interaction analysis revealed potential ligand-receptor interactions within this niche involving Timp1 or Ackr1

**Fig. 4:**
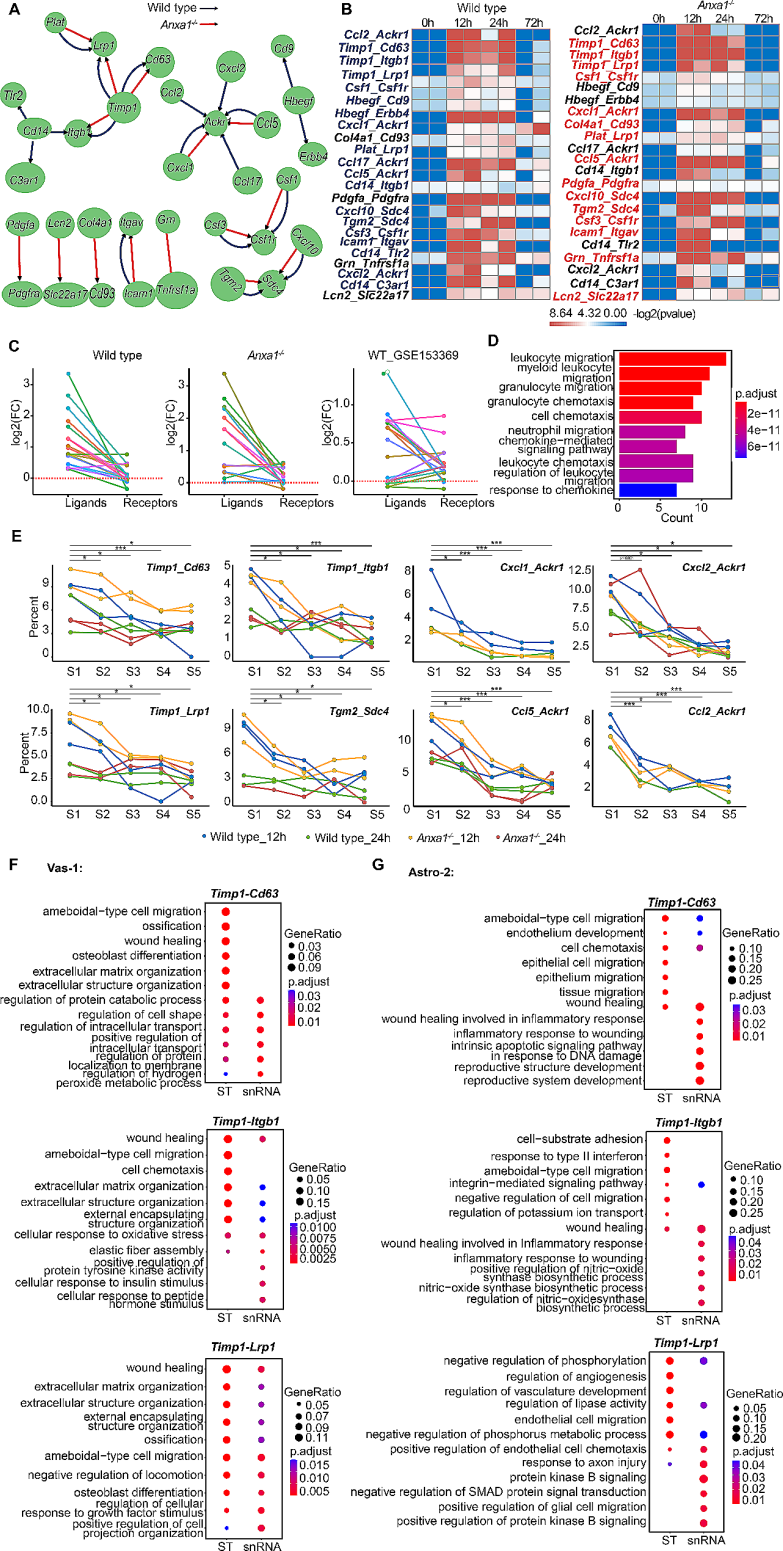
Crosstalk among microglia, astrocytes, and vascular components within the pathological microstructure. (**A**) Network diagram of ligand-receptor pairs that are specifically activated in wild type or *Anxa1*^−/−^ mice at 12- and 24-hour time points (referred to as responsive-LRs). Each node in the diagram represents either a ligand or receptor, with the node from which the arrow originates representing the ligand and the node to which the arrow points representing the receptor. (**B**) Heatmap displaying the -log_2_ (*p*-value) of the activity of responsive ligand-receptor (LR) pairs in wild type and knockout (*Anxa1*^−/−^) mice at different time points. LR pairs were identified as responsive if they showed significant changes in activity compared to baseline levels. The heatmap uses a color-coding scheme to indicate whether a responsive LR is specific to wild type (blue) or *Anxa1*^−/−^ (red) mice. The heatmap provides a visual representation of the changes in activity of specific LR pairs in wild type and *Anxa1*^−/−^ mice over time. (**C**) Fold change values of genes composed of responsive ligand-receptor (LR) pairs. Fold change values were calculated in wild type and *Anxa1*^−/−^ mice at 12- and 24-hour time points compared to 0- and 72-hour time points. Additionally, fold change values were calculated in a publicly available bulk RNA-seq dataset comparing LPS-treated wild type mice to PBS-treated wild type mice. The genes included in the analysis are those that are composed of the responsive LR pairs identified in the previous analysis. The figure provides a visual representation of the changes in gene expression in response to LPS treatment in wild type and *Anxa1*^−/−^ mice, as well as in a separate dataset. (**D**) The enriched GO BP-terms in the genes composed of responsive-LRs in wild type mice. Top ten GO BP-terms are shown. (**E**) Distribution of ligand-receptor (LR) co-localization spot percent in different spot groups. The spots were selected based on the presence of any of the nine labels (Astro-1, Astro-2, Micro-1, Micro-2, Neuron, OligoD, OPC, Vas-1, and Cdkn1a^+^ Serpina3n^+^ OligoD) and grouped according to the combination of labels present in each ST dataset. LR co-localization spot percent in each spot group was calculated and plotted across eight ST datasets from mice at 12- and 24-hour time points. The top five spot types with the highest percentages are shown in the figure and labeled as S1, S2, S3, S4, and S5. The LR co-localization spot percent between S1 and S2, S3, S4, and S5 is compared by T-test (paired samples, one-tailed); statistical significance is indicated by asterisks (*P < 0.05; ***P < 0.001). In each subgraph, S1 represents the spot group composed of Micro-2, Astro-2, and Vas-1 labels. The figure provides insights into the co-localization patterns of LR pairs within specific cell types and across different ST datasets. (**F**-**G**) The GO BP-terms that are enriched in the up-regulated genes between Vas-1 cells (spots) with and without the ligand-receptor pair(LR) (F) and between Astro-2 cells (spots) with and without the LR (G). A cell or a spot with a LR pair means that the two genes composed the LR are co-expressed in the cell or spot. The analysis was performed to identify the biological processes that are associated with the up-regulated genes between cells with and without LR pairs. The top six enriched GO BP-terms are shown for both ST data and snRNA-seq data. The figure provides insights into the biological processes that are affected by the presence or absence of LR pairs in Vas-1 and Astro-2 cells

To gain insights into cellular interactions, we conducted an analysis of co-localization scores for over 2,000 ligand-receptor gene pairs and identified 19 pairs activated pairs in wild-type brains and 15 pairs in *Anxa1*^*−/−*^ mice at both the 12-hour and 24-hour time points following LPS challenge, with 11 pairs exhibiting activation in both groups. Notably, among the identified pairs, we observed the presence of three pairs involving *Timp1* and *Cd14*, five pairs with *Ackr1*, and two pairs with *Csf1r* and *Sdc4* (Fig. 4A). Intriguingly, an in-depth analysis of the snRNA-seq data uncovered high expression levels of *Timp1* in the Astro-2 and Vas-1 clusters, while its receptor exhibited a more widespread expression pattern across multiple cell types. This intriguing expression pattern implies a potential regulatory interaction between Astro-2 and Vas-1 cells that may exert an influence on other cellular entities. Likewise, we observed a predominant expression of *Ackr1* in neurons and Vas-1 cells, accompanied by the noteworthy presence of its ligands in Astro-2 and Micro-2 subtypes, suggesting the existence of bidirectional interactions. Furthermore, our analysis revealed the expression of *Cd14* and its receptors within the Micro-2 subtype, indicating the potential involvement of self-regulatory mechanisms within this specific cluster. In addition, the expression of *Csf1* and *Cxcl10*, along with their respective receptors, was detected in both Astro-2 and Micro-2 subtypes, implying the possibility of facilitating mutual regulation or autoregulation processes. Moreover, we observed a predominant expression of *Csf1* in the Astro-2 subtype and *Cdkn1a*^*+*^*Serpina3n*^+^ OligoD cell population, Intriguingly, the receptor for *Csf1*, namely *Csf1r*, exhibited specific localization in Micro-2 subtype. This finding suggests a potential regulatory relationship, wherein Astro-2 cells and *Cdkn1a*^*+*^*Serpina3n*^+^ OligoD population may govern the activity of Micro-2 cells through the *Csf1-Csf1r* ligand-receptor pathway. Additionally, our analysis revealed the specific presence of *Cxcl10* and *Sdc4* in both Astro-2 and Micro-2 subtypes, further implicating their potential involvement in either mutual regulation or autoregulation mechanisms. (Supplementary Figure S4A). Following the initial analysis, spatial co-expression analysis was performed to validate the co-expression patterns of these ligand-receptor genes in both wild type and *Anxa1*^*−/−*^ mice at 12-hour and 24-hour time points post-challenge. Notably, certain gene pairs, including *Col4a1-Cd93* and *Pdgfa-Pdgfra*, maintained their co-expression status until 72-hour time point in wild type mice. Conversely, in *Anxa1*^*−/−*^ mice, the co-expression of some gene pairs diminished before the 24-hour mark. Collectively, no significant disparity in gene co-expression was observed at the 12-hour post-challenge time point between the two mouse groups (Fig. 4B). Subsequently, we assessed the differential gene expression levels of these ligand-receptor pairs and observed that majority of genes in the brains of both wild-type and *Anxa1*^*−/−*^ mice exhibited upregulation at both the 12-hour and 24-hour time points following LPS challenge. To confirm the robustness of these findings, we cross-validated our results by analyzing the GSE153369 dataset, which yielded consistent outcomes (Fig. 4C). Furthermore, a GO enrichment analysis on the upregulated genes shed light on their involvement in immune cell migration pathways, including leukocyte, myeloid leukocyte, granulocyte migration, and chemotaxis (Fig. 4D). Consequently, we further investigated the spatial distribution of these genes at the 12-hour and 24-hour time points, specifically focusing on the expression patterns within clusters such as Astro-2, Micro-2, and Vas-1 (collectively referred to as V1A2M2). Notably, hub genes such as *Timp1* and *Ackr1* exhibited highly expression levels within these clusters. Similarly, the *Tgm2-Sdc4* pair demonstrated elevated expression within the same clusters. These findings imply that the intricate interactions among these cell types within the V1A2M2 structure are potentially facilitated through the involvement of *Timp1* and *Ackr1* genes (Fig. 4E).

Our analysis of snRNA-seq data revealed that *Ackr1* exhibited predominant expression (Supplementary Figure S4A). However, further investigation through spatial transcriptomic analysis unveiled its presence within the V1A2M2 region. To validate its expression in Vas-1, we performed an examination of *Ackr1* across 46 mixed cell type groups. Our findings confirmed its expression in Vas-1, as illustrated in the fourth box plot of Supplementary Figure S4B. Additionally, to explore the potential role of the hub gene *Timp1* and its associated receptors in the V1A2M2 region, we conducted a GO analysis focusing on the upregulated genes present in Vas-1 and Astro-2 cells expressing *Timp1*. Notably, we identified *Cd63*, *Itgb1*, and *Lrp1* as receptor genes associated with *Timp1* in this context. The analysis revealed a significant enrichment of pathways related to wound healing and amoeboid cell migration in both the snRNA-seq and Stereo-seq datasets, suggesting that Vas-1 and Astro-2 cell populations may play a crucial role in inflammatory repair processes and potentially facilitate the migration of Micro-2 cells towards Vas-1 through *Timp1*-mediated signaling (Fig. 4F and G). This study hypothesizes that Vas-1 and Astro-2 populations, characterized by their expression of the *Timp1* receptor gene, may play a role in the regulation of inflammatory repair. Furthermore, considering the well-established evidence of amoeboid-type movement exhibited by activated microglia in previous investigations [53–56], it is plausible to suggest that the expression of *Timp1* in Vas-1 and Astro-2 cells potentially facilitates the migration of Micro-2 cells. Spatial transcriptomic analysis revealed that the expression of ligand-receptor genes within the V1A2M2 region exhibited a peak at 12 h following LPS stimulation, followed by a decrease by the 24-hour time point, and eventual disappearance by 72 h. Notably, no significant disparities in gene expression were observed between wild type and *Anxa1*^*−/−*^ mice (Supplementary Figure S4C). Furthermore, the hub gene *Cd14* and its associated receptors, primarily localized in microglial spots, indicated a potential self-regulatory mechanism of Micro-2 cells through the involvement of these genes during the inflammatory response (Supplementary Figure S5A). Subsequent GO analysis focused on the activation of Micro-2 cells mediated by *Cd14* unveiled significant associations with immune cell migration processes. Notably, these processes encompassed the regulation of inflammatory response, neutrophil migration, and myeloid leukocyte migration, suggesting a potential role for Micro-2 cells in facilitating immune cell movement. Consequently, the heightened activity of Micro-2 cells observed in the vicinity of blood vessels may be attributable to their migratory behavior (Supplementary Figures S5C, S5B, and S5D). Simultaneous analysis revealed the widespread expression of the ligand-receptor pair *Cxcl10-Sdc4* across diverse cellular populations within the spatial transcriptome spots. Notably, this pair exhibited specific and high expression levels in Astro-2 and Micro-2 cells. GO enrichment analysis conducted on the co-expressed genes within these clusters indicated an augmented migration capacity in Micro-2 cells, alongside an elevated immune response in Astro-2 cells due to the influence of *Cxcl10-Sdc4* pair. This co-expression pattern was found to be associated with inflammatory responses and signaling pathways related to cytokines or type II interferon. These findings underscore the crucial role played by *Cxcl10-Sdc4* in the inflammatory dynamics of Astro-2 and Micro-2 cells (Supplementary Figures S5E and S5F).

Our findings highlight the upregulation and co-expression of genes belonging to ligand-receptor pairs, including hub genes *Timp1* and *Ackr1*. These genes play crucial roles in processes such as immune cell migration, chemotaxis, inflammatory responses, and signaling pathways associated with cytokines or type II interferon. Notably, increased expression of these genes was observed within the specialized pathological niche V1A2M2 in the brains of both wild type and *Anxa1*^*−/−*^ mice at 12 h and 24 h following LPS challenge. The absence of *Anxa1* resulted in a more rapid decline of the protective structure composed of Aastro-2/Micro-2/Vas-1 during the reactive phase, potentially leading to reduced survival.

**Fig. 5:**
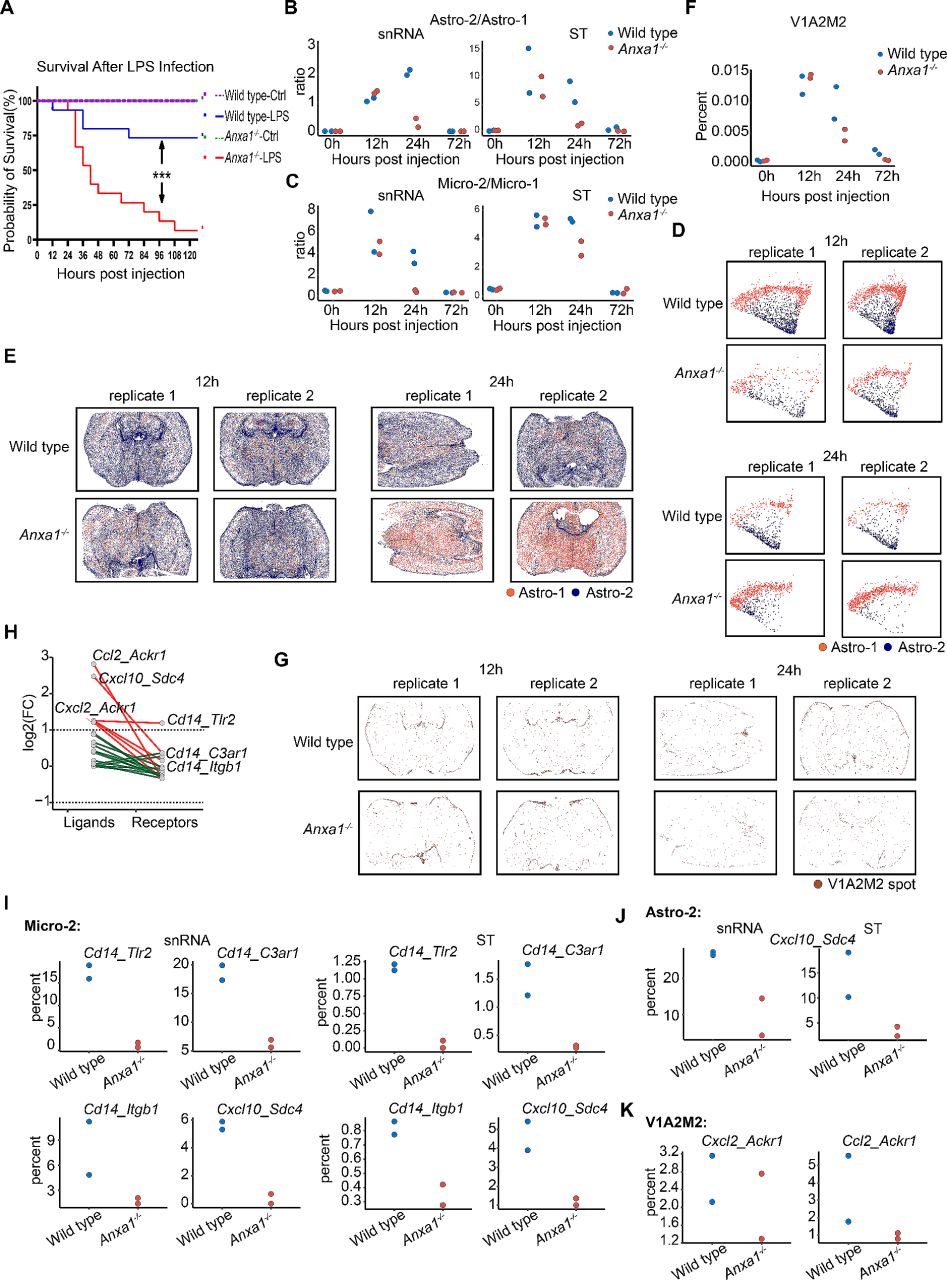
Role of *Anxa1* in the pathogenesis of pathological microstructure formation. (**A**) Survival Analysis of murine model through intraperitoneal administration of lipopolysaccharide (LPS) in wild type and *Anxa1*^−/−^ group. Graphical representation is as follows: wild type control mice receiving saline (purple dashed line), wild type mice administered lipopolysaccharide (LPS) (blue solid line), *Anxa1*^−/−^ control mice receiving saline (green dashed line), and *Anxa1*^−/−^ mice administered LPS (red solid line). On the survival plot, the x-axis signifies the duration of post-injection observation in hours, while the y-axis denotes the survival percentage. A statistical significance, indicated by three asterisks, corresponds to a *P* value of less than 0.001. (**B**-**C**) The ratio between cells (spots) of different cell subtypes across time periods. Specifically, it shows the ratio of Astro-2 cells (spots) with Astro-1 cells (spots) (**B** and the ratio of Micro-2 cells (spots) with Micro-1 cells (spots) (**C**). Each dot in the figure represents the ratio calculated from a mouse, and the color of the dot indicates the genotype of the mouse. The ratio calculation was performed on two different types of data: “snRNA” represents the ratio calculated in the snRNA-seq data, while “ST” indicates that the ratio was calculated in the ST data. (**D**) UMAP plot showing Astro-2 and Astro-1 cells from 12- and 24-hour mice in the snRNA-seq data. The Astro-2 and Astro-1 cells are indicated by different colors in the plot. (**E**) Spatial map showing the distribution of Astro-2 and Astro-1 labeled spots in the ST datasets from 12- and 24-hour mice. The Astro-2 and Astro-1 labeled spots are represented by different colors on the map, indicating their respective locations. (**F**) Percents of Vas-1, Astro-2, and Micro-2 labeled spots in the ST data from different time periods. Each dot in the figure represents a mouse, and the color of the dot represents the mouse genotype. The percentages of Vas-1, Astro-2, and Micro-2 labeled spots were calculated for each mouse and are displayed in the figure. (**G**) Spatial map of Vas-1, Astro-2, and Micro-2 labeled spots in the ST datasets from 12- and 24-hours mice. (**H**) Fold change values of genes composed of responsive ligand-receptor pairs between 24-hour wild type mice and 24-hour *Anxa1*^−/−^ mice. The genes that showed a fold change of two times or more, either up-regulated (red) or down-regulated (green), were identified. The corresponding ligand-receptor pairs associated with these genes were highlighted. (**I**-**J**) Percentages of Micro-2 cells (spots) with the LR in 24-hour wild type mice and 24-hour *Anxa1*^−/−^ mice (**I**), as well as the percentages of Astro-2 cells (spots) with *Cxcl10-Sdc4* in 24-hour wild type mice and 24-hour *Anxa1*^−/−^ mice. (**K**) Percentages of Vas-1, Astro-2, and Micro-2 labeled spots with the ligand-receptor pair in 24-hour wild type mice and 24-hour *Anxa1*^−/−^ mice. Each dot in the figure represents a mouse, and the color of the dot indicates the mouse genotype. A spot with a ligand-receptor pairs indicates that the two genes composing the LR are co-localized in that particular spot

To elucidate the significance of ANXA1 during this process, we conducted a survival analysis to compare the outcomes between LPS stimulated *Anxa1*^*−/−*^ and wild type mice. Notably, the survival rates of *Anxa1*^*−/−*^ mice were notably lower than those of wild type mice, underscoring the protective role of *Anxa1* in the context of LPS-induced sepsis (Fig. 5A). Subsequently, we performed a comparative analysis to assess the impact of *Anxa1* on the inflammatory response in the brain following endotoxin stimulation. Through a comparative analysis of the ratios of Astro-2 to Astro-1 in brain tissues from both wild type and *Anxa1*^*−/−*^ mice, we identified a significant increase in the proportion of Astro-2 at 12 h post-stimulation, with consistent trends observed in both snRNA-seq and Stereo-seq datasets. However, at 24 h post-stimulation, the levels of Astro-2 in *Anxa1*^*−/−*^ mice exhibited a significant decrease compared to those in wild type mice, indicating a substantial down-regulation linked to the knockout of *Anxa1* (Fig. 5B). A similar trend was observed in the proportional shifts between Micro-2 and Micro-1 cells, which exhibited significant increases in both mouse types at 12 h post-stimulation. However, at 24 h, there was a slight reduction in the proportion of Micro-2 cells specifically in *Anxa1*^*−/−*^ mice (Fig. 5C). Subsequent analyses conducted using snRNA-seq and spatial transcriptome visualization approaches confirmed that the decline in Astro-2 at 24 h post-stimulation in *Anxa1*^*−/−*^ mice was significantly more pronounced compared to the wild-type group, establishing a direct correlation with the genetic knockout of the *Anxa1* gene (Fig. 5D and E). We analyzed the temporal expression patterns of a 130-gene module, referred to as V1A2M2, in the brain tissues of wild type and *Anxa1*^*−/−*^ mice. This module was identified as the highest scoring cluster in our analysis. At 12 h post-LPS stimulation, we observed a significant upregulation of these genes in both mouse groups. However, by the 24-hour time point, the extent of gene downregulation in *Anxa1*^*−/−*^ mice was notably more pronounced compared to wild type mice (Fig. 5F). These findings were further confirmed through spatial transcriptome visualization, providing additional support for the observed patterns (Fig. 5G). Additionally, at 24 h following LPS challenge, we observed significantly lower expression levels of certain ligand-receptor pairing genes in *Anxa1*^*−/−*^ mice compared to wild type mice, which could potentially be attributed to the decreased numbers of the specialized pathological niche V1A2M2 (Fig. 5H). Further analysis of cell percentages associated with these genes showed significant decreases in the proportions of Micro-2 cells expressing *Cd14* and its receptors, as well as Astro-2 and Micro-2 cells expressing *Cxcl10-Sdc4*. Similarly, the proportions of Astro-2/Micro-2/Vas-1 cells expressing *Ccl2-Ackr1* also showed a significant decline. These findings suggest that the knockout of *Anxa1* influenced the expression of *Cd14*, *Cxcl10-Sdc4*, and *Ccl2-Ackr1*, ultimately leading to diminished proportions of the V1A2M2 structure (Fig. 5I, J and K).

## Discussion

SAE, a severe complication of sepsis, poses a considerable challenge in clinical practice due to its intricate pathophysiology and substantial implications for morbidity and mortality. Given the potential confounding factors in neurological assessment within the intensive care unit (ICU) setting, a comprehensive and multimodal approach is necessary for accurate diagnosis and effective management. Standard diagnostic measures encompass clinical evaluation, metabolic tests, and, in selected cases, neuroimaging and electroencephalography. SAE is defined by a complex array of neurological symptoms that manifest as a consequence of systemic infection. However, the precise mechanisms that underlie the development of septic encephalopathy remain incompletely understood, posing a significant challenge to the advancement of accurate diagnostic approaches and the formulation of effective therapeutic interventions [57]. By employing keyword burst detection, it was revealed that neuroinflammation, blood-brain barrier (BBB) integrity, and mitochondria dysfunction have emerged as prominent areas of research focus [58]. Notably, significant progress has been made in elucidating the underlying mechanisms of neuroinflammation, which have provided valuable insights into the pathogenesis of SAE. A prevailing hypothesis posits that dysregulation of the immune response, characterized by an imbalance between pro-inflammatory and anti-inflammatory pathways, plays a pivotal role in the development and progression of SAE. Uncontrolled neuroinflammation and ischemic injury have been identified as key contributors to the development of brain dysfunction in SAE. Additionally, investigations have delved into the intricate interplay between SAE and peripheral immunity, aiming to unravel the complex mechanisms underlying the systemic impact of sepsis on brain function. Emerging scientific literature has brought attention to the pivotal role of various mediators in the disruption of the BBB during sepsis. Notably, recent studies propose that therapeutic interventions aimed at modulating microglial activation, ameliorating endothelial cell dysfunction, and preventing BBB permeability hold considerable promise in improving the clinical outcomes of SAE [59].

The *Anxa1* gene, recognized for its anti-inflammatory properties within the nervous system, has gained attention as a potential modulator in SAE. Previous studies have provided evidence of the protective effects of *Anxa1* in models of neuroinflammation, indicating its involvement in modulating the progression of the disease. In a recent study, researchers made a finding wherein the administration of recombinant ANXA1 (rANXA1) exhibited robust protective effects on the BBB in animal models. This intervention was associated with a reduction in BBB disruption, brain edema, and the loss of endothelial junction proteins [60]. Furthermore, parallel studies have demonstrated the critical role of ANXA1 in brain physiology, particularly its involvement in the endothelium of the BBB. These investigations have emphasized the potential of ANXA1 to exert protective actions in the context of neuroinflammatory, neurovascular, and metabolic diseases [61]. Additionally, ANXA1 levels have been observed to be higher in the brains of individuals with Alzheimer’s disease (AD) as well as in animal models during the initial stages of the disease. ANXA1 has the ability to enhance the breakdown of amyloid-beta (Aβ) through neprilysin in N2a cells, and it also assists in the engulfment of Aβ by microglia. These effects are mediated by the involvement of FPRL1 receptors. Moreover, ANXA1 acts as an inhibitor by reducing the release of inflammatory substances by microglia stimulated by Aβ [62]. Researchers have found that the interaction between SIRT5 and ANXA1 at K166 causes the desuccinylation of ANXA1, leading to a reduction in its SUMOylation level. This process prevents ANXA1 from being recruited to the membrane and secreted outside the cell, resulting in an overactive response from microglia, increased production of proinflammatory cytokines and chemokines, and ultimately damage to neuronal cells in cases of ischemic stroke [63]. In another study, researchers aimed to investigate whether the ANXA1 tripeptide could hinder the activation of NLRP3 inflammasome, which in turn could prevent the activation of microglial and memory deficits related to the hippocampal-dependent [64, 65].

The specific involvement of *Anxa1* in SAE, particularly regarding the progression of the disease over time, remains largely unexplored. This knowledge gap emphasizes the necessity for comprehensive research that investigates the temporal patterns of inflammatory responses and examines the potential role of *Anxa1* in these processes. To address this concern, our study utilized a comprehensive experimental approach by employing a mouse model of SAE induced by intraperitoneal injection of LPS [66]. This mouse model enables us to investigate the development of SAE at critical time intervals, with special emphasis on the initial phases of the disease. We employed advanced techniques such as snRNA-seq and Stereo-seq [67–70] to gain a comprehensive understanding of the cellular composition and diversity following LPS stimulation in the brain. SnRNA-seq enables in-depth analysis of individual cells, allowing us to explore the heterogeneity within the brain. This is essential to comprehend intricate neurological mechanisms and identify specific cell types that may contribute to disease processes [71]. Although snRNA-seq offers detailed cellular information, it lacks spatial context. Spatial transcriptomics, on the other hand, provides data on the precise locations of different cells within the tissue. This spatial information is particularly significant in the brain, as the organization and location of cells are closely tied to their functions [72, 73]. By combining these technologies, we can gain a more comprehensive understanding of the functional aspects of the brain. Our approach provides a more holistic perspective by analyzing both the individual cellular responses and their spatial organization within the complex brain microenvironment. This allows us to capture the diversity of cell types and their spatial distribution in a more comprehensive manner.

**Fig. 6:**
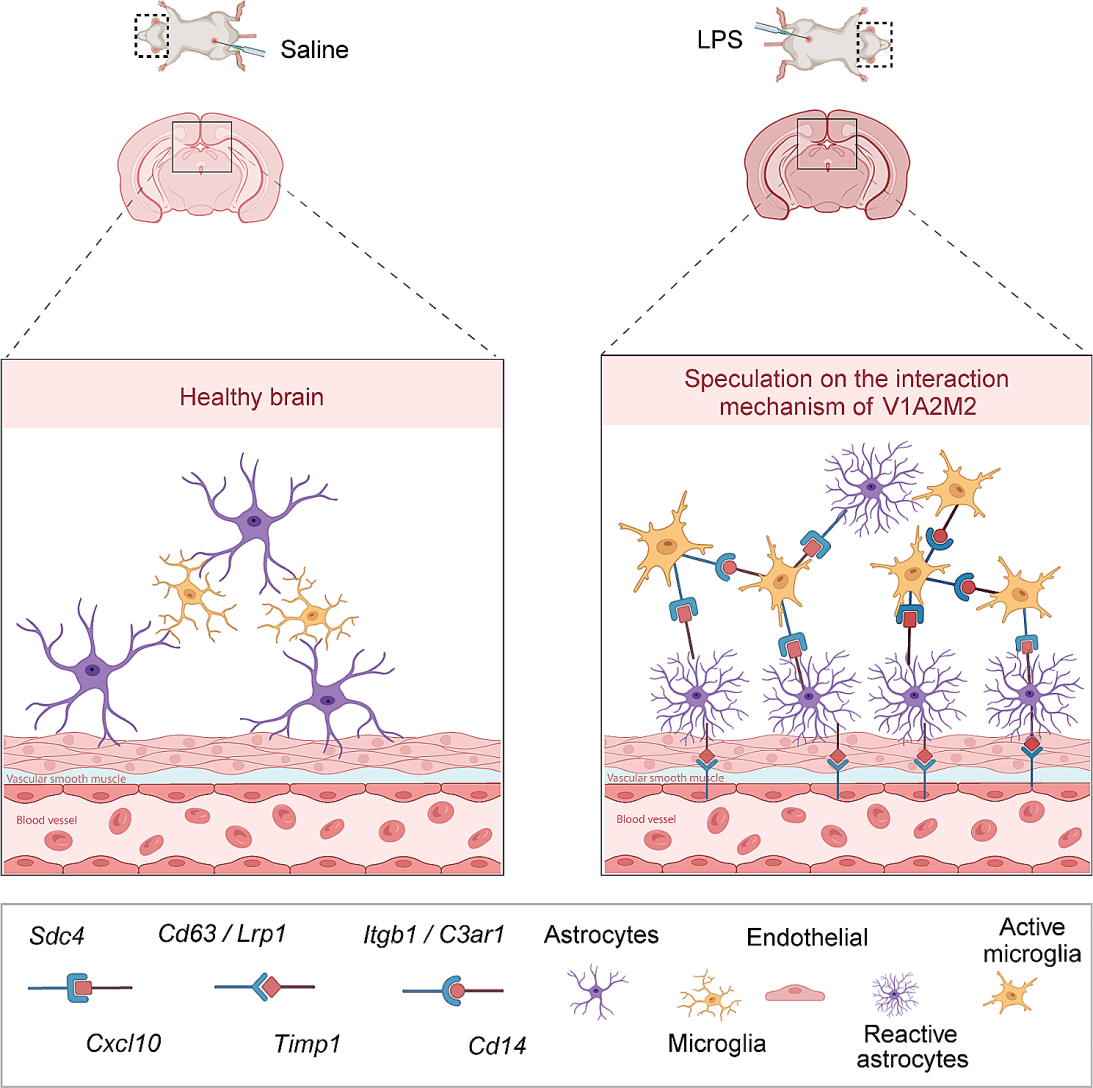
Hypothesized model of astroglial and microglial perivascular network interactions. Glial cell activation and interaction following intraperitoneal injection of lipopolysaccharide (LPS) were compared to the control group. After intraperitoneal injection, microglia and astrocytes were observed to be activated, proliferate, and enlarge in the peritoneal cavity. These activated glial cells were found to aggregate around blood vessels, forming a structure referred to as V1A2M2. The activated glial cells present in the V1A2M2 structure may act by activating specific receptor ligands. Activated astrocytes in the structure may interact through *Timp1-Cd63* and *Timp1-Lrp1*, while activated microglia may engage in self-regulation through *Cd14-Itgb1* and *Cd14-C3ar1*. This response signifies the reaction to intracranial inflammation initiated by peripheral inflammation

In our study, detailed findings in the results section uncovered several important discoveries regarding the activation of glial cells, their spatial distribution, and the distinct response observed in knockout mice. The data obtained from snRNA-seq demonstrated a significant increase in activated microglial cells and astrocytes at 12 h and 24 h after LPS injection. These findings were further supported by spatial transcriptome sequencing, which provided spatial context to the observed cellular activation in both wild-type and *Anax1* knockout mice. The timing of glial cell activation following LPS stimulation can vary. Previous studies have reported that changes in glial cells can be detected as early as 3 h after LPS injection [74]. Another study documented an elevation in specific activation markers in the brain 4 h after LPS administration [75]. Additionally, one study examined mice that were sacrificed 24 h after intracerebroventricular (ICV) LPS injection for further analysis [76]. Activated microglia have been found to provide neuroprotection against brain injuries. For example, a study demonstrated that mice injected with LPS experienced decreased neuronal cell death and lesion volumes following experimental brain injury [77]. This neuroprotective effect is thought to be achieved by reducing inhibitory axosomatic synapses. LPS stimulation can induce neuroinflammation in microglia by activating certain pathways and suppressing others, thereby inhibiting autophagosome formation [78]. Such neuroinflammation is associated with the development of neurodegenerative diseases. LPS stimulation of microglia primarily triggers a pro-inflammatory response, activating other glial cells and peripheral immune response, which collectively contributes to the establishment of a neuroinflammatory phenotype [79]. Furthermore, spatial analysis uncovered a unique pattern of activated glial cell distribution around blood vessels at 12 h and 24 h, suggesting a targeted response in that area. This finding contrasts with a previous study, which established that glial cells have the capability to both dilate and constrict blood vessels, a mechanism involved in neurovascular coupling. This important discovery emphasizes the close interaction between glial cells and blood vessels [80]. Analysis of ligand profiles unveiled a simultaneous increase in specific receptor-ligand pairs at 12 h and 24 h, suggesting potential signaling pathways implicated in glial cell activation (Fig. 6). Further examination of these receptor-ligand pairs showed that both Astro-2 and Vas-1 upregulated *Timp1-Itgb1*/*Cd63* after 24 h, which is linked to wound healing and amoeboid cell migration. A study demonstrated that TIMP-1 promotes hypermigration of primary dendritic cells infected with *Toxoplasma* through CD63-ITGB1-FAK signaling [81]. This finding implies a potential involvement of the *Timp1-Itgb1*/*Cd63* interaction in microglia migration. In another study, it was discovered that *TIMP1* interacts with the CD63/ITGB1 complex, leading to the activation of downstream FAK signaling. This activation, in turn, reduces RhoA activation and promotes the stability of endothelial cell structure by preventing F-actin depolymerization. These findings imply that the interaction between *TIMP1* and *ITGB1/CD63* may contribute to maintaining the integrity of the blood-brain barrier, which is vital for neuroprotection [82]. Additionally, we detected an increase in the expression of *ACKR1* receptor and chemokines in the CNS. Although the role of ACKRs in the CNS is multifaceted and extends beyond inflammation control, there are specific cases where *CXCR7/ACKR3* has been shown to regulate T cell trafficking by modulating the internalization of *CXCL12* at CNS endothelial barriers. ACKR1 plays a critical regulatory role in chemokine biology by capturing, scavenging, or transporting chemokines, thus modulating their availability and signaling through conventional chemokine receptors [83]. An example of such a chemokine is *CCL2*, also known as monocyte chemoattractant protein 1 (MCP-1), which attracts monocytes and microglia to areas of brain inflammation. Notably, it has been associated with the recruitment of microglia and macrophages in chronic traumatic encephalopathy [84]. The interaction between chemokines like *CCL2* and *ACKR1* can modulate the inflammatory response in the brain, which is a crucial component of neuroinflammation. The increase in *CD14* and Toll-Like Receptors 2 (TLR2) observed in this study confirms previous findings that indicates their involvement in microglial activation induced by fibrillar Aβ [85]. Additionally, this finding is consistent with the positive regulation of defense responses identified in the GO analysis conducted in this experiment. Notably, when comparing wild-type and knockout mice, noticeable differences were primarily observed in the quantity and arrangement of activated astrocytes at 24 h, accompanied by the reduced expression of specific receptor-ligand pairs (*Ccl2-Ackr1*, *Cd14-Tlr2*). These findings suggest that the knockdown of *Anxa1* results in a reduce in V1A2M2 structure, decreased astro-2 count, less micro-2 recruitment, and an attenuated inflammatory response in microglial cells. Collectively, these findings enhance our understanding of the temporal and spatial dynamics in glial cell activation following LPS injection. They also provide insights into potential signaling mechanisms and highlight distinctions between wild-type and *Anax1* knockout mice. However, there are a few limitations in this study that need to be addressed. Firstly, the number of samples at each time point is limited. Additionally, this study only focuses on the effects of LPS challenge on the brain in mice. In future studies, it would be beneficial to investigate the impact of sepsis on other tissues and organs, such as the intestine, heart, liver, kidney and spleen etc. Finally, the use of spatial omics methods, such as spatial proteomics, can be employed to directly examine the expression changes of important regulatory proteins in various brain regions at different time points following LPS stimulation. Furthermore, employing spatial metabolomics can enable the examination of alterations in metabolite levels within various regions of the brain in mice, specifically those that are temporally regulated following LPS stimulation.

In conclusion, our study highlights the importance of considering the temporal dynamics of neuroinflammatory responses in murine model of SAE. By integrating snRNA-seq and Stereo-seq techniques, we have identified a special pathological niche composed of Astro-2 and Micro-2 and Vas-1 cells, accompanied by the upregulation of specific ligand-receptor pairs. These findings suggest potential involvement of these mechanisms in the development of SAE or the mortality associated with *Anxa1* knockdown. Future investigations focusing on the interplay between *Anxa1*, different anti-inflammatory pathways, and their contributions to the progression of SAE could contribute further to our understanding of this complex disease.

### Electronic supplementary material

Below is the link to the electronic supplementary material.

**Supplementary Material 1: Supplementary Figure 1**

**Supplementary Material 2: Supplementary Figure 2**

**Supplementary Material 3: Supplementary Figure 3**

**Supplementary Material 4: Supplementary Figure 4**

**Supplementary Material 5: Supplementary Figure 5**

**Supplementary Material 6: Supplementary Figure 6**

**Supplementary Material 7: Supplementary Table 1**. Display of gene signatures for Astro-1, Astro-2, Micro-1, Micro-2, Neuron, OligoD, OPC, Vas-1, and Cdkn1a^+^ Serpina3n^+^ OligoD from the snRNA-seq data

**Supplementary Material 8: Supplementary Table 2**. Display of the features and counts number per cell of snRNA-seq

**Supplementary Material 9: Supplementary Table 3**. Display of DEGs across Astro-2 vs. Astro-1 clusters in snRNA-seq data

**Supplementary Material 10: Supplementary Table 4**. Display of DEGs across Micro-2 vs. Micro-1 in snRNA-seq data

**Supplementary Material 11: Supplementary Table 5**. Display of primer sequences used in PCR experiments

**Supplementary Material 12: Supplementary Table 6**. Module of 130 genes

## Data Availability

No datasets were generated or analysed during the current study.

## Abbreviations

BBB: Blood-brain barrier
CID: Coordinate identity
CNS: Central nervous system
DEGs: Differentially expressed genes
H&E: Hematoxylin and eosin
ICV: Intracerebroventricular
IHC: Immunohistochemical
IP: Intraperitoneal injection
LPS: Lipopolysaccharide
LRs: Ligand-receptor pairs
MIDs: Molecular identifiers
OCT: Optimal cutting temperature
OPC: Oligodendrocyte precursor cells
PBS: Phosphate buffered saline
PCR: Polymerase chain reaction
SAE: Sepsis-associated encephalopathy
snRNA-seq: Single nucleus RNA sequencing
ST: Spatial transcriptome sequencing
UMAP: Uniform Manifold Approximation and Projection
UMIs: Unique molecular identifiers
