# Supplementary material for: Integrating single-nucleus RNA sequencing and spatial transcriptomics to elucidate a specialized subpopulation of astrocytes, microglia and vascular cells in brains of mouse model of lipopolysaccharide-induced sepsis-associated encephalopathy
Source: J Neuroinflammation. 2024 Jul 3;21:169. doi: 10.1186/s12974-024-03161-0 (PMC11223438; doi:10.1186/s12974-024-03161-0)
Supplement: Supplementary file 1 — Supplementary Material 1: Supplementary Figure 1 [file 12974_2024_3161_MOESM1_ESM.docx]

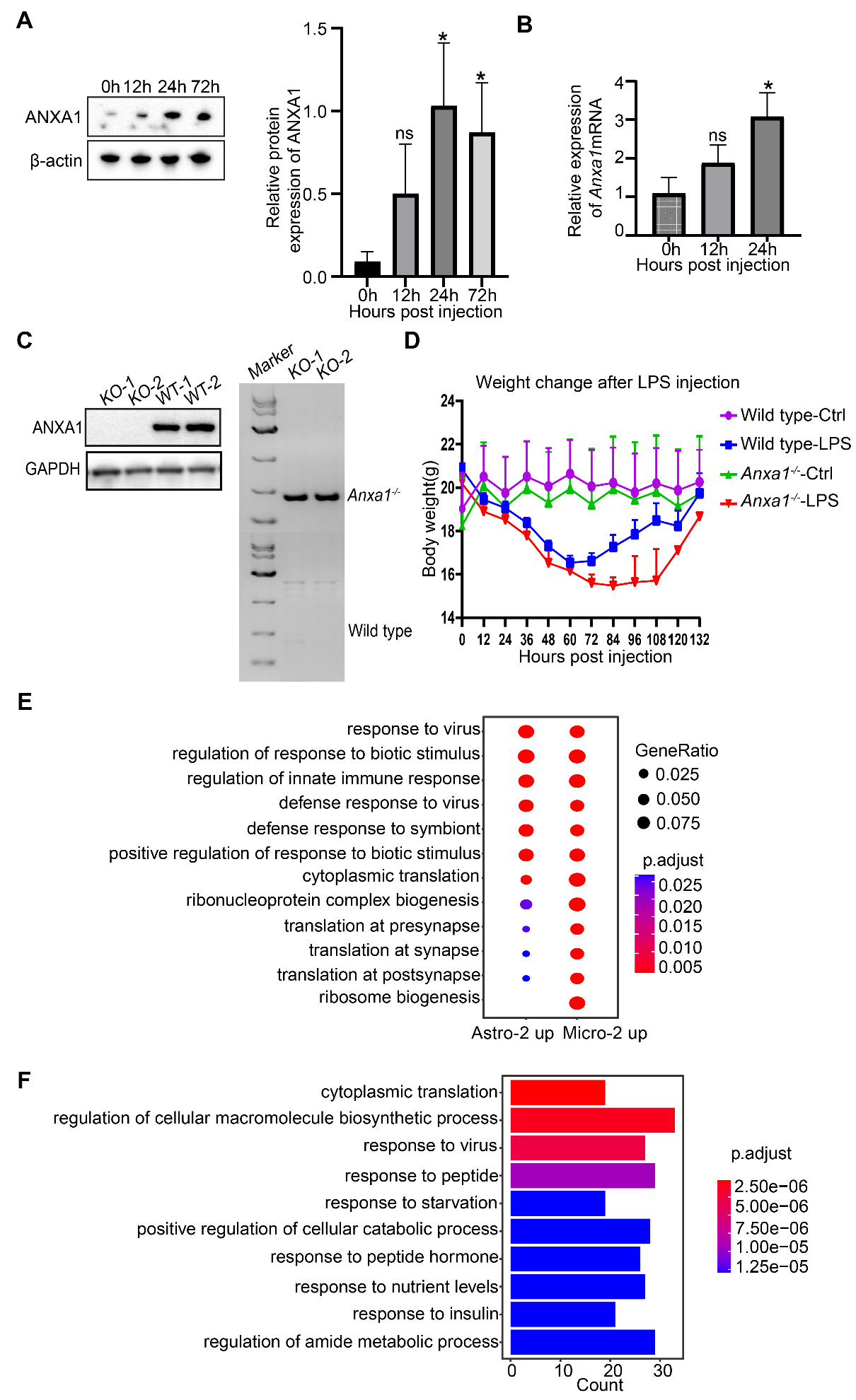


**Supplementary Figure 1.**

(A-B) Western blot and quantitative fluorescence PCR analyses were conducted to assess the expression levels of Anxa1 in the brains of wild type mice following LPS stimulation at 0 h, 12 h, 24 h, 72 h. * *P* < 0.05. Data are expressed as means SD; n 3 mouse/ group. * *P* < 0.05, 24 h group compared with 0 h group; ns *P* > 0.05, 12 h group compared with 0 h group.

(C) Genotype validation of mouse samples: (Left) Western blot (WB) analysis: four samples were analyzed, with the left two lanes representing wild-type (wild type) mouse brain tissue, and the right two lanes knockout (*Anxa1*^-/-^) mouse brain tissue. At 37 kDa, *Anxa1*^-/-^ mice exhibited one band, whereas wild type mice showed no bands. GAPDH bands were present in all four lanes. (Right) Polymerase chain reaction (PCR) validation: The upper part of the graph depicts *Anxa1*^-/-^ band validation, while the lower part validates wild type bands. The left two wells contain *Anxa1*^-/-^ mouse tail gene samples, and the right two wells wild type mouse tail gene samples. *Anxa1*^-/-^ bands were observed in the left two wells without wild type bands, and vice versa in the right two wells.

(D) Body weight changes in wild type and *Anxa1*^-/-^ mice following LPS stimulation: This graph represents the body weight trajectories over time post-injection. Four groups are illustrated: wild type mice injected with saline (purple curve), wild type mice injected with lipopolysaccharide (LPS) (blue curve), *Anxa1*^-/-^ mice injected with saline (green curve), and *Anxa1*^-/-^ mice injected with LPS (red curve). The x-axis denotes hours post-injection, while the y-axis represents the body weight of the mice in grams.

(E) The enriched GO BP-terms (biological process terms) in the DEGs between Astro-2 and Astro-1, as well as between Micro-2 and Micro-1 in the snRNA-seq data, were determined. The top six GO BP-terms for the up-regulated genes in Astro-2 and Micro-2 are respectively identified and presented.

(F) The GO BP-terms that were enriched in the DEGs between Oligodendrocytes from wild type and *Anxa1*^-/-^ mice at 12- or 24-hours, and at 0- or 72-hours, were identified. The top ten GO BP-terms for the up-regulated genes are displayed.
