# Supplementary material for: Integrating single-nucleus RNA sequencing and spatial transcriptomics to elucidate a specialized subpopulation of astrocytes, microglia and vascular cells in brains of mouse model of lipopolysaccharide-induced sepsis-associated encephalopathy
Source: J Neuroinflammation. 2024 Jul 3;21:169. doi: 10.1186/s12974-024-03161-0 (PMC11223438; doi:10.1186/s12974-024-03161-0)
Supplement: Supplementary file 2 — Supplementary Material 2: Supplementary Figure 2 [file 12974_2024_3161_MOESM2_ESM.docx]

**
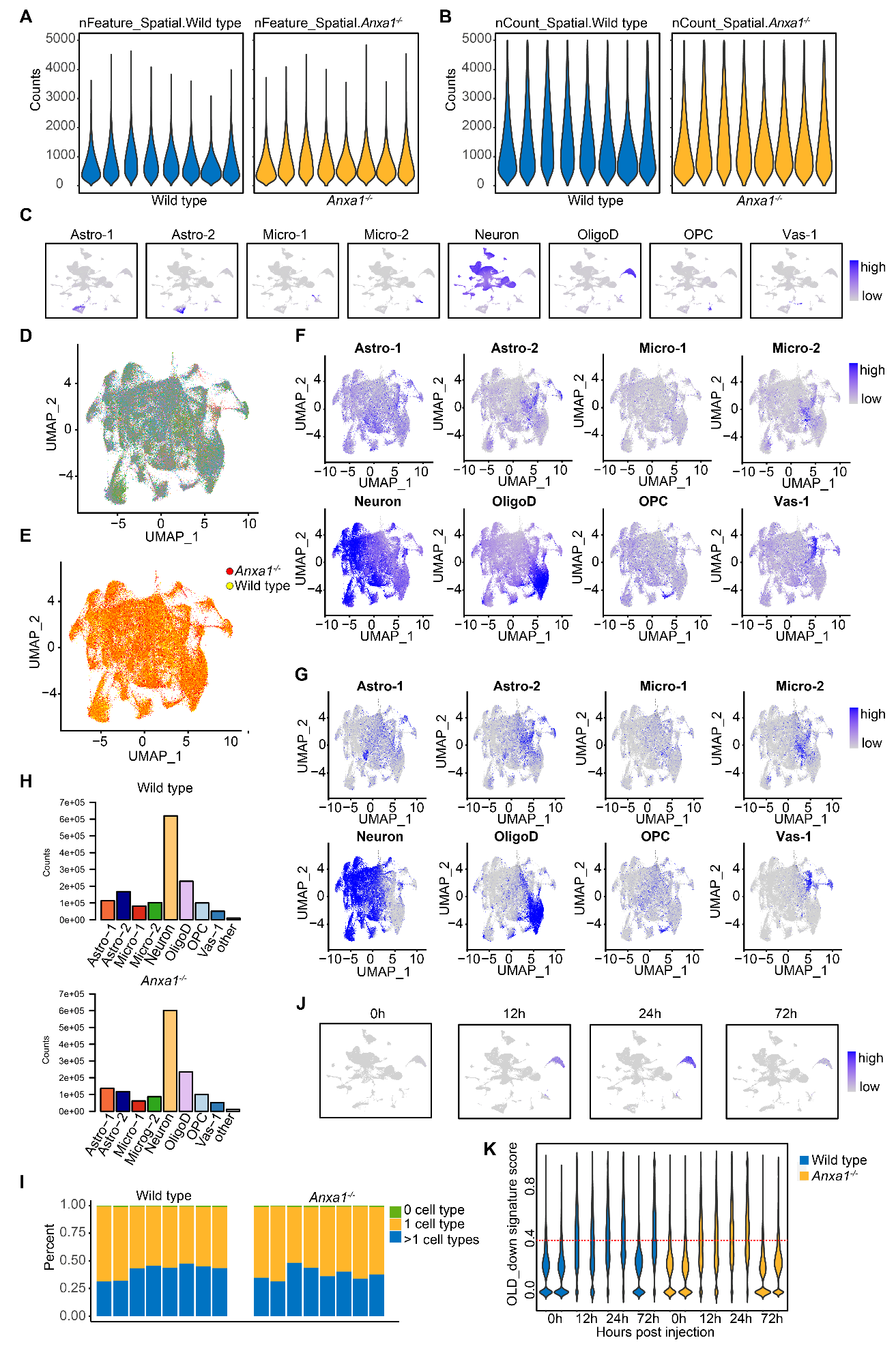
**

**Supplementary Figure 2.**

(A-B) Distribution of gene counts (A) and UMI counts (B) of spots across the ST data.

(C) Visualization of signature scores for the eight cell types in the UMAP plot of the snRNA-seq data.

(D) The UMAP plot displays 320,000 randomly selected spots from all sixteen ST data. The spots are color-coded based on the respective ST data they belong to.

(E) UMAP plot of the 320,000 spots, which are color-coded based on the ST data from the wild type mice (yellow) and *Anxa1*^-/-^ mice (red).

(F) Visualization of the signature scores for the eight cell types in the UMAP plot containing the 320,000 spots.

(G) Visualization of spots labeled with the eight cell types in the UMAP plot comprising the 320,000 spots.

(H) The number of spots with the eight assigned cell type labels in the ST data of wild type mice or *Anxa1*^-/-^ mice. Spots that do not fall into any of the eight cell type labels (Astro-1, Astro-2, Micro-1, Micro-2, Neuron, OligoD, OPC, and Vas-1) are labeled as "other".

(I) Relative proportions of spots assigned with no cell type label, one cell type label, and more than one cell type labels in the ST data.

(J) Visualization of Cdkn1a^+^ Serpina3n^+^ OligoD signature score in cells from different time periods in the snRNA-seq data.

(K) Distribution of the Cdkn1a^+^ Serpina3n^+^ OligoD signature score in Oligodendrocyte spots across the ST data. The Oligodendrocyte spots with a score above 0.4 were also assigned Cdkn1a^+^ Serpina3n^+^ OligoD labels.
