# Supplementary material for: Integrating single-nucleus RNA sequencing and spatial transcriptomics to elucidate a specialized subpopulation of astrocytes, microglia and vascular cells in brains of mouse model of lipopolysaccharide-induced sepsis-associated encephalopathy
Source: J Neuroinflammation. 2024 Jul 3;21:169. doi: 10.1186/s12974-024-03161-0 (PMC11223438; doi:10.1186/s12974-024-03161-0)
Supplement: Supplementary file 3 — Supplementary Material 3: Supplementary Figure 3 [file 12974_2024_3161_MOESM3_ESM.docx]

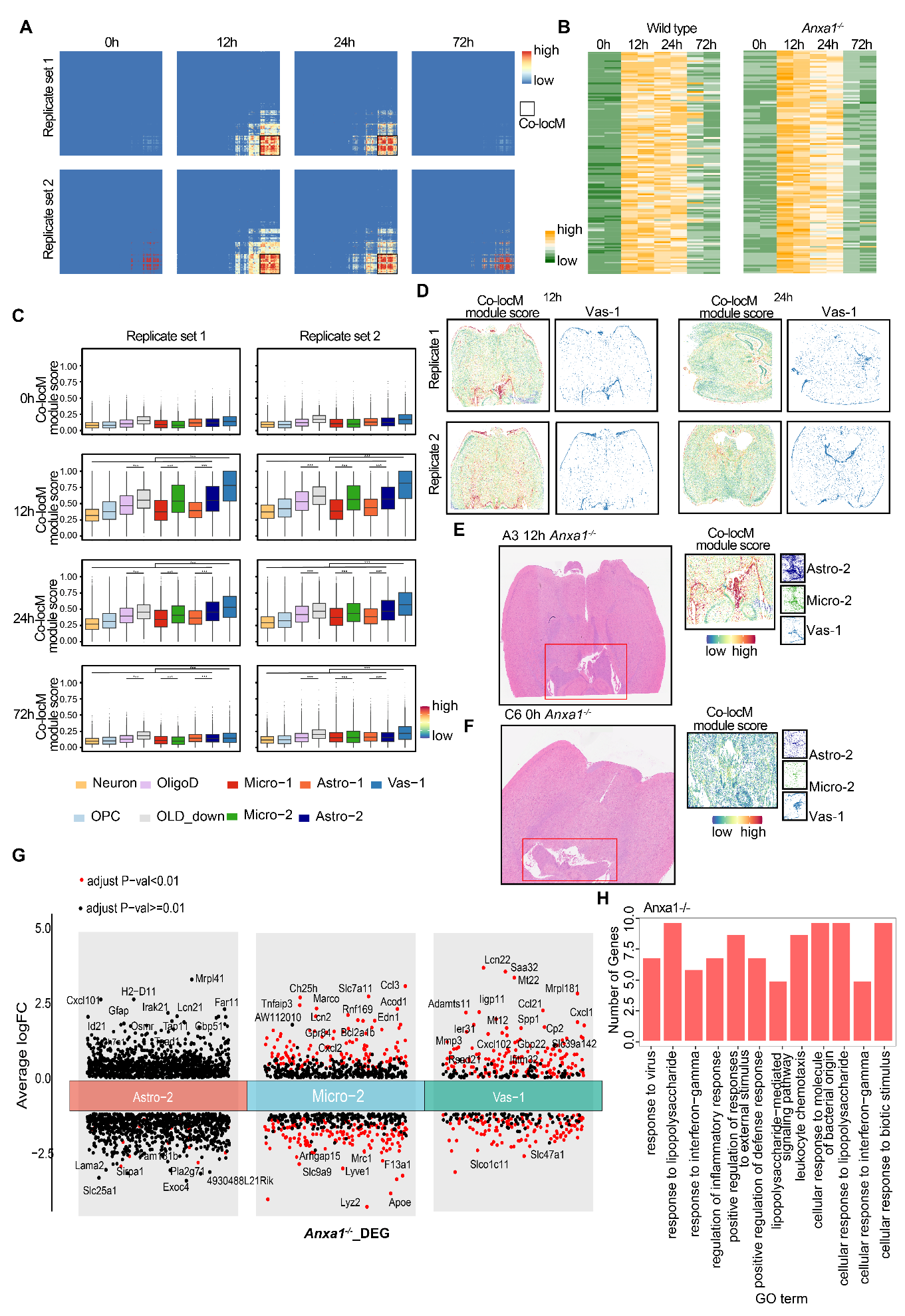


**Supplementary Figure 3.**

(A) Heatmap illustrating the Co-locM’ s genes in the ST data of *Anxa1*^-/-^ mice from different time periods after LPS treatment.

(B) Heatmap showing expression patterns of Co-locM’ s genes in wild type and *Anxa1*^-/-^ mice from different time periods after LPS treatment.

(C) Distribution of the Co-locM module score in spots assigned with different cell type labels in the ST data of *Anxa1*^-/-^ mice from different time periods. The boxplot for each spot type is color-coded based on its label type. T tests (unpaired samples, two-tailed) were conducted between different spot types, and the *p*-value or the maximum *p*-value of a test set was reported. Significance levels are indicated as follows: *** *P* < 0.001.

(D) Spatial distribution of the Co-locM module score and the spatial map of Vas-1 in the ST data obtained from *Anxa1*^-/-^ mice at 12- and 24-hour time points.

(E-F) Spatial maps of Astro.2, Micro-2, and Vas-1 spots in two different regions from *Anxa1*^-/-^ mice. The first region, as a Co-locM hot spot (E), is from a 12-hour mouse and represents a region where all three cell types are co-localized. The second region is a paired region from a 0-hour mouse (F). The maps illustrate the distribution and co-localization of these three cell types in each region.

(G) DEG analysis showing up- and downregulated genes across Astro-2, Micro-2 and Vas-1 clusters in snRNA-seq data of *Anxa1*^-/-^ mice. An adjusted *P* value < 0.01 is indicated in red, while an adjusted *P* value ≥ 0.01 is indicated in black. DEG, differentially expressed genes.

(H) The GO terms obtained by performing GO enrichment analysis on the top 30 genes of Astro-2, Micro-2 and Vas-1 clusters based on the log_2_ FC value in the snRNA-seq data of *Anxa1*^-/-^ mice.
