# Supplementary material for: Integrating single-nucleus RNA sequencing and spatial transcriptomics to elucidate a specialized subpopulation of astrocytes, microglia and vascular cells in brains of mouse model of lipopolysaccharide-induced sepsis-associated encephalopathy
Source: J Neuroinflammation. 2024 Jul 3;21:169. doi: 10.1186/s12974-024-03161-0 (PMC11223438; doi:10.1186/s12974-024-03161-0)
Supplement: Supplementary file 4 — Supplementary Material 4: Supplementary Figure 4 [file 12974_2024_3161_MOESM4_ESM.docx]

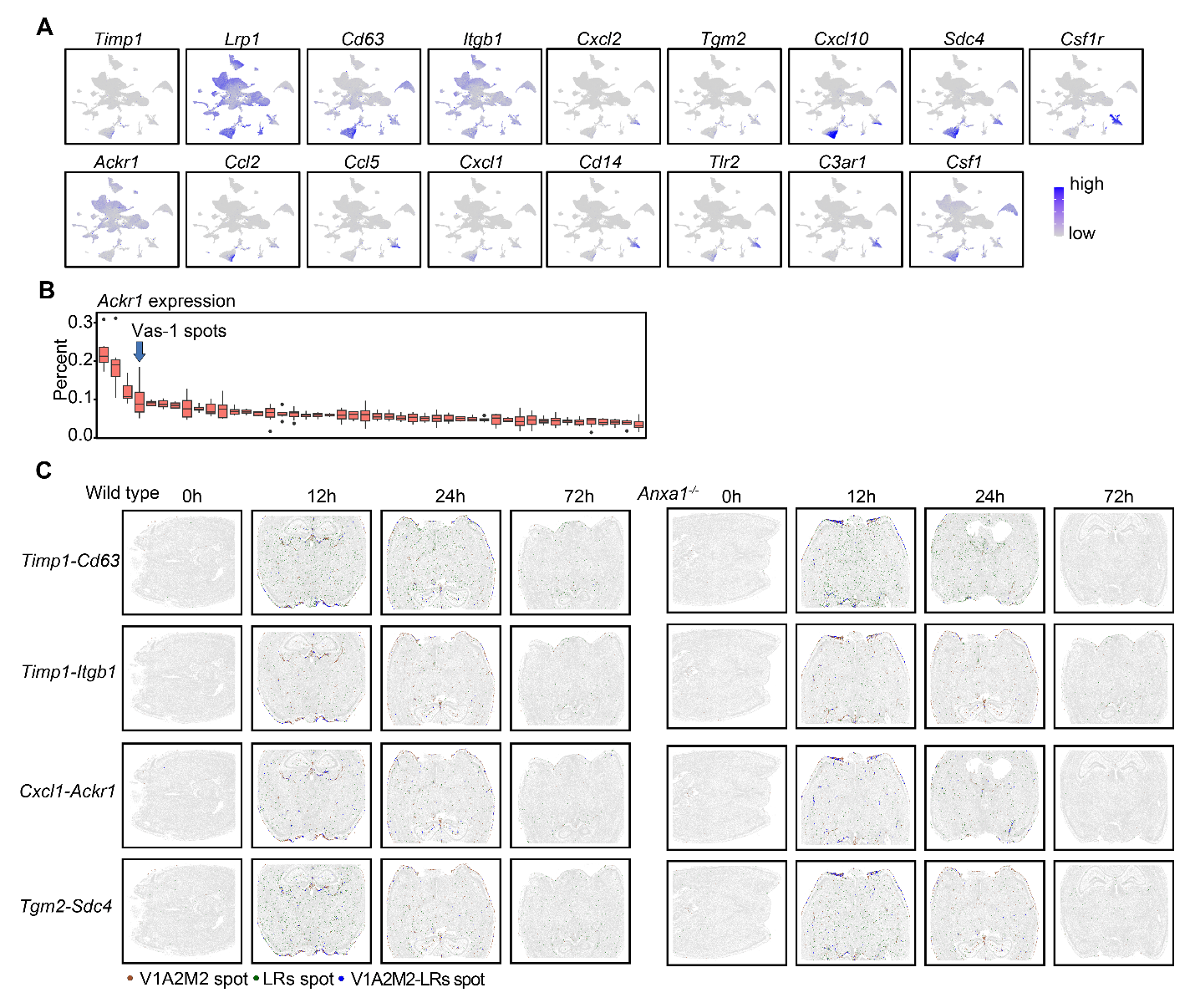


**Supplementary Figure 4.**

(A) Expression patterns of genes composed of responsive ligands and receptors in the UMAP plot of the snRNA-seq data.

(B) Percent of *ACKR1* expression in the 46 spot groups. Each spot group is represented by a boxplot, which displays the distribution of *Ackr1* expression spot percent across eight spatial ST datasets from mice at 12- and 24-hour time points. The spot group that exclusively contains the Vas-1 label is specifically indicated. *Ackr1* expression spot percent represents the proportion of spots within each spot group that exhibit expression of the *Ackr1* gene.

(C) Spatial map illustrating the distribution of the ligand-receptor pairs co-localization spots (LRs spot) and Vas-1, Astro-2 and Micro-2 co-localization spots (spots with Vas-1, Astro-2 and Micro-2 labels, V1A2M2 spot) in the ST datasets from wild type and *Anxa1*^-/-^ mice at various time periods. The figure allows for comparisons between wild type and *Anxa1*^-/-^ mice and across different time periods. Ligand-receptor pairs co-localization spots represent spots where two genes composing a ligand-receptor pairs are co-expressed, while V1A2M2 co-localization spots represent spots that possess all three labels- Vas-1, Astro-2, and Micro-2. The figure provides insights into the co-localization patterns of specific cell types and ligand-receptor pairs across different ST datasets.
