# Supplementary material for: Integrating single-nucleus RNA sequencing and spatial transcriptomics to elucidate a specialized subpopulation of astrocytes, microglia and vascular cells in brains of mouse model of lipopolysaccharide-induced sepsis-associated encephalopathy
Source: J Neuroinflammation. 2024 Jul 3;21:169. doi: 10.1186/s12974-024-03161-0 (PMC11223438; doi:10.1186/s12974-024-03161-0)
Supplement: Supplementary file 5 — Supplementary Material 5: Supplementary Figure 5 [file 12974_2024_3161_MOESM5_ESM.docx]

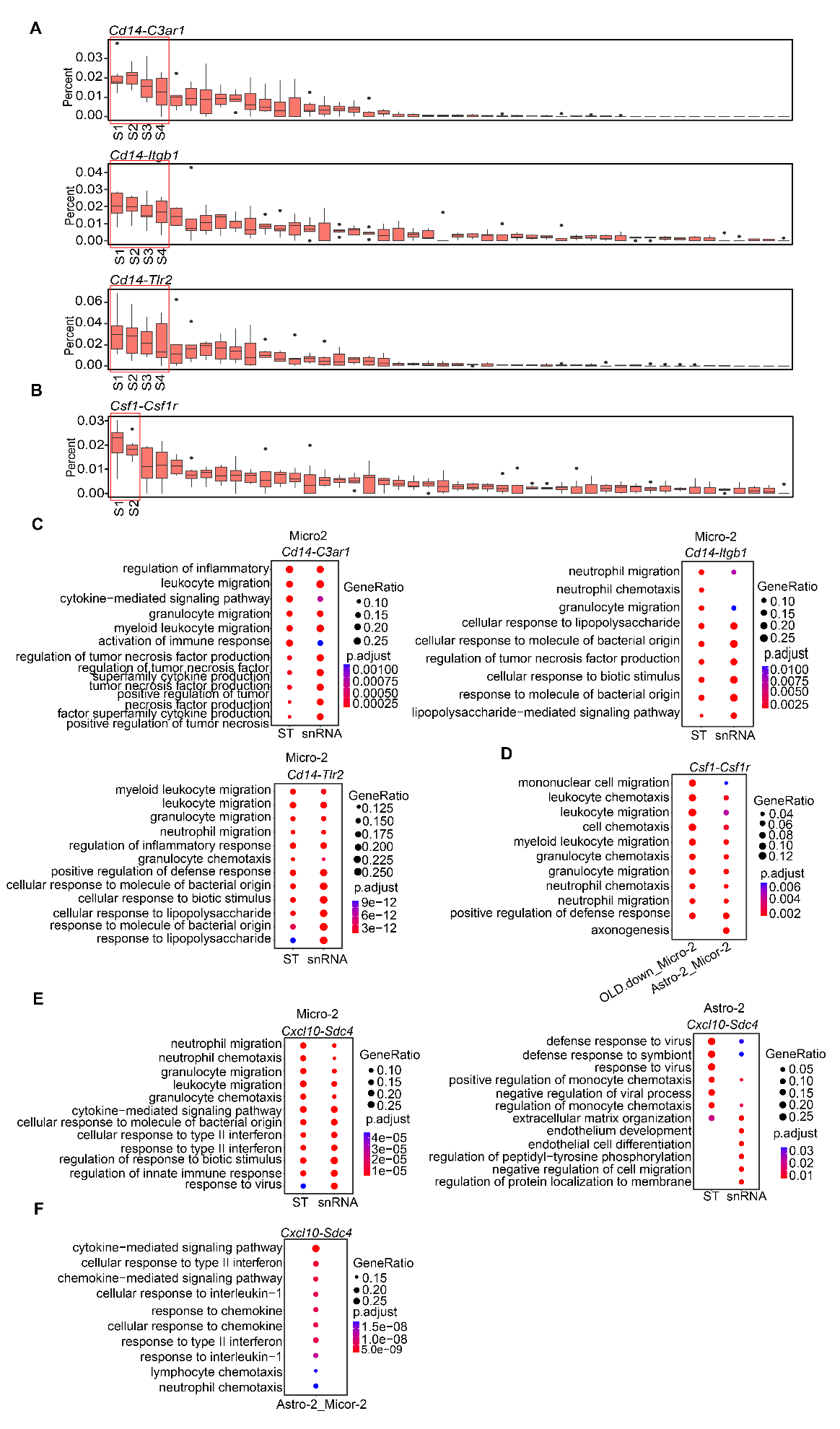


**Supplementary Figure 5.**

(A) Distribution of ligand-receptor pair co-localization spots for *Cd14-C3ar1*, *Cd14-Itgb1*, and *Cd14-Tlr2* in 46 spot groups. Each boxplot represents the percentage of ligand-receptor pair co-localization spots in a spot group across eight ST datasets from mice at 12 and 24 hours. In the *Cd14-C3ar1* subgraph, spot group S1 contains spots labeled as Micro-2, spot group S2 contains spots labeled as Micro-1 and Micro-2, spot group S3 contains spots labeled as OligoD, Micro-2, and Cdkn1a^+^ Serpina3n^+^ OligoD, and spot group S4 contains spots labeled as Neuron, Micro-1, and Micro-2. In the *Cd14-Itgb1* subgraph, spot group S1 contains spots labeled as Micro-2, spot group S2 contains spots labeled as Micro-2, Astro-2, and Vas-1, spot group S3 contains spots labeled as OligoD, Micro-2, Astro-2, and Cdkn1a^+^ Serpina3n^+^ OligoD, and spot group S4 contains spots labeled as OligoD, Micro-2, and Cdkn1a^+^ Serpina3n^+^ OligoD. In the *Cd14-Tlr2* subgraph, spot group S1 contains spots labeled as Micro-2, spot group S2 contains spots labeled as OligoD, Micro-2, and Cdkn1a^+^ Serpina3n^+^ OligoD, spot group S3 contains spots labeled as Micro-1 and Micro-2, and spot group S4 contains spots labeled as Neuron, Micro-1, and Micro-2.

(B) Distribution of ligand-receptor pair co-localization spots for *Csf1-Csf1r* in 46 spot groups. Each boxplot represents the percentage of *Csf1-Csf1r* co-localization spots in a spot group across eight ST datasets from mice at 12 and 24 hours. Spot group S1 contains spots labeled as OligoD, Micro-2 and Cdkn1a^+^ Serpina3n^+^ OligoD, spot group S2 contains spots labeled as OligoD, Micro-2, Astro-2 and Cdkn1a^+^ Serpina3n^+^ OligoD.

(C) The GO BP-terms that are enriched in the up-regulated genes between Micro-2 cells (spots) with and without ligand-receptor pair for *Cd14-C3ar1*, *Cd14-Itgb1*, and *Cd14-Tlr2*. A cell or a spot with a ligand-receptor pair pair means that the two genes composed the ligand-receptor pair are co-expressed in the cell or spot. The analysis was performed to identify the biological processes that are associated with the up-regulated genes between cells with and without ligand-receptor pairs. The top six enriched GO BP-terms are shown for both ST data and snRNA-seq data.

(D) The GO BP-terms enriched in the up-regulated genes between the spots with and without *Csf1-Csf1r*. Two distinct groups of up-regulated genes were identified: one in spots labeled as Cdkn1a^+^ Serpina3n^+^ OligoD and Micro-2, and another in spots labeled as Astro-2 and Micro-2. For each up-regulated gene group, the top six GO BP-terms were identified and are presented in the figure. These GO BP-terms indicate the biological processes that are significantly associated with the DEGs in each respective group.

(E) GO BP-terms enriched in the up-regulated genes between Micro-2 cells (spots) with and without *Cxcl10-Sdc4*, as well as between Astro-2 cells (spots) with and without *Cxcl10-Sdc4*. Differential gene expression analysis identified up-regulated genes in each group of spots. For each group, the top six GO BP-terms were identified and are presented in the figure. These GO BP-terms indicate the biological processes that are significantly associated with the DEGs in each respective group.

(F) The GO BP-terms enriched in the up-regulated genes between Astro-2 and Micro-2 labeled spots with and without *Cxcl10-Sdc4*. Differential gene expression analysis identified up-regulated genes in each group of spots. The top ten GO BP-terms enriched in the up-regulated genes are shown in the figure. These GO BP-terms indicate the biological processes that are significantly associated with the DEGs between the two groups of spots.
