# Supplementary material for: Integrating single-nucleus RNA sequencing and spatial transcriptomics to elucidate a specialized subpopulation of astrocytes, microglia and vascular cells in brains of mouse model of lipopolysaccharide-induced sepsis-associated encephalopathy
Source: J Neuroinflammation. 2024 Jul 3;21:169. doi: 10.1186/s12974-024-03161-0 (PMC11223438; doi:10.1186/s12974-024-03161-0)
Supplement: Supplementary file 6 — Supplementary Material 6: Supplementary Figure 6 [file 12974_2024_3161_MOESM6_ESM.docx]

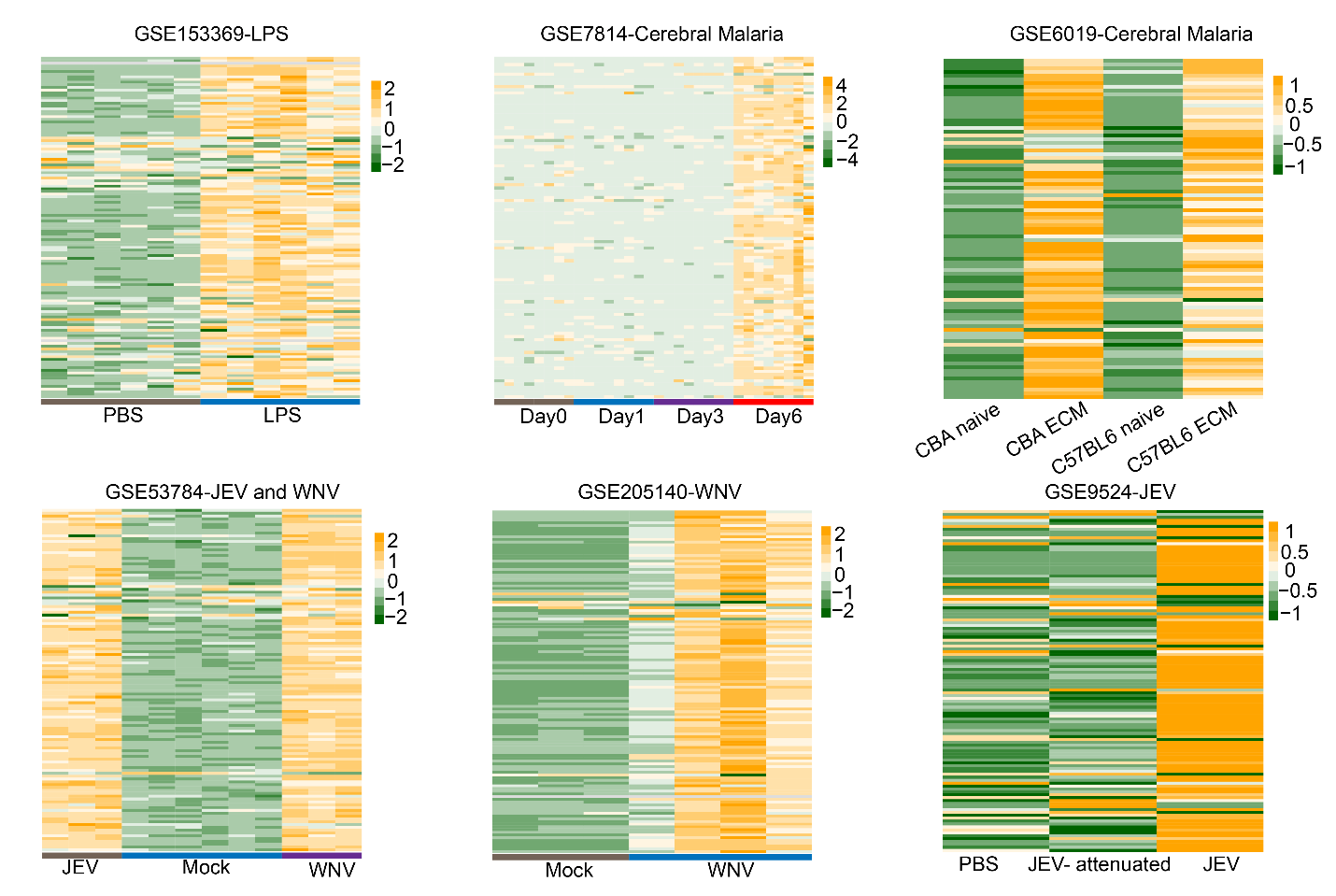


**Supplementary Figure 6.**

The heatmap illustrates the expression of genes in the Co-locM across publicly available mouse brain gene expression datasets. The subgraphs within the heatmap represent different experimental conditions and treatments.

In the "GSE153369-LPS" subgraph, "PBS" and "LPS" indicate mice treated with PBS and LPS, respectively.

In the "GSE7814-Cerebral Malaria" subgraph, "Day0", "Day1", "Day3", and "Day6" represent different time points after mice were infected with P. Berghia ANKA (PbA).

In the "GSE6019-Cerebral Malaria" subgraph, "CBA naive" and "CBA ECM" represent control CBA mice and CBA mice that developed experimental cerebral malaria (ECM), respectively. Similarly, "C57BL6 naive" and "C57BL6 ECM" represent control C57BL6 mice and C57BL6 mice that developed ECM.

In the "GSE53784-JEV and WNV" subgraph, "JEV", "Mock", and "WNV" indicate mice infected with Japanese encephalitis virus (JEV), mock virus, and West Nile virus (WNV), respectively.

In the "GSE205140-WNV" subgraph, "Mock" and "WNV" represent mice infected with mock virus and WNV, respectively.

In the "GSE9524-JEV" subgraph, "PBS", "JEV-attenuated", and "JEV" represent mice with no JEV infection, attenuated JEV infection, and JEV infection, respectively.
